# PKM2 regulates endothelial cell junction dynamics and angiogenesis via ATP production

**DOI:** 10.1038/s41598-019-50866-x

**Published:** 2019-10-21

**Authors:** Jesús Gómez-Escudero, Cristina Clemente, Diego García-Weber, Rebeca Acín-Pérez, Jaime Millán, José A. Enríquez, Katie Bentley, Peter Carmeliet, Alicia G. Arroyo

**Affiliations:** 1 Vascular Pathophysiology, Centro Nacional de Investigaciones Cardiovasculares (CNIC). Melchor Fernández Almagro 3, 28029 Madrid, Spain; 2 Tumour Biology Department, Barts Cancer Institute, John´s Vane Centre, Queen Mary´s University of London. Charterhouse Sq, EC1M 6BQ London, UK; 3 Centro de Investigaciones Biológicas (CIB-CSIC). Ramiro de Maeztu 9, 28040 Madrid, Spain; 4 Centro de Biología Molecular Severo Ochoa, Consejo Superior de Investigaciones Científicas (CSIC), Universidad Autónoma de Madrid, 28049 Madrid, Spain; 5 Myocardial Pathology Areas, Centro Nacional de Investigaciones Cardiovasculares (CNIC). Melchor Fernández Almagro 3, 28029 Madrid, Spain; 6 Computational Biology Laboratory, Beth Israel Deaconess Medical Center, Harvard Medical School, Boston, MA USA; 7 Cellular Adaptive Behaviour Laboratory, Rudbeck Laboratories, Department of Immunology, Genetics and Pathology, Uppsala University, Uppsala, Sweden; 8 Laboratory of Angiogenesis and Vascular Metabolism, Center for Cancer Biology, Vlaams Instituut voor Biotechnologie (VIB), B-3000 Leuven, Belgium; 9 Laboratory of Angiogenesis and Vascular Metabolism, Center for Cancer Biology, Department of Oncology, University of Leuven, B-3000 Leuven, Belgium; 10 State Key Laboratory of Ophthalmology, Zhongsan Ophthalmic Center, Sun Yat-Sen University, Guangzhou, China

**Keywords:** Collective cell migration, Angiogenesis, Adherens junctions

## Abstract

Angiogenesis, the formation of new blood vessels from pre-existing ones, occurs in pathophysiological contexts such as wound healing, cancer, and chronic inflammatory disease. During sprouting angiogenesis, endothelial tip and stalk cells coordinately remodel their cell-cell junctions to allow collective migration and extension of the sprout while maintaining barrier integrity. All these processes require energy, and the predominant ATP generation route in endothelial cells is glycolysis. However, it remains unclear how ATP reaches the plasma membrane and intercellular junctions. In this study, we demonstrate that the glycolytic enzyme pyruvate kinase 2 (PKM2) is required for sprouting angiogenesis *in vitro* and *in vivo* through the regulation of endothelial cell-junction dynamics and collective migration. We show that PKM2-silencing decreases ATP required for proper VE-cadherin internalization/traffic at endothelial cell-cell junctions. Our study provides fresh insight into the role of ATP subcellular compartmentalization in endothelial cells during angiogenesis. Since manipulation of EC glycolysis constitutes a potential therapeutic intervention route, particularly in tumors and chronic inflammatory disease, these findings may help to refine the targeting of endothelial glycolytic activity in disease.

## Introduction

Angiogenesis, the formation of new blood vessels from pre-existing ones, occurs in pathophysiological contexts such as wound healing, cancer and chronic inflammatory disease. In sprouting angiogenesis, endothelial cells (ECs) receive a stimulus (usually VEGFA) which triggers the angiogenic response by the selection and specification of the endothelial tip cell^1^. This cell migrates toward the stimulus followed by the stalk cells, defined by a balanced Dll4/VEGFA signaling^2^. A fine-tuned coordination of diverse cellular responses is necessary at this initial step, including reorganization of endothelial cell junctions, tip-cell migration to generate characteristic cytoskeletal protrusions called filopodia, and proliferation and lumenization by stalk cells. Impairment in any of these processes prevents productive angiogenesis and the formation of a functional vascular plexus^3–5^. A shared feature of all these endothelial cell responses is the need for energy in the form of ATP^6,7^.

The predominant ATP generation route in ECs is glycolysis^8^, providing both the ATP required for cell function and the intermediate metabolites that feed into other metabolic pathways for macromolecule biosynthesis^9,10^. During sprouting angiogenesis, glycolysis is boosted in the endothelial tip cell, and angiogenesis is blunted by the genetic deletion or pharmacological inhibition of glycolytic enzymes like PFKFB3^11–13^. Recent dissection of the impact of distinct metabolic routes in endothelial cells has revealed that glycolysis impacts endothelial cell migration (in addition to proliferation), whereas fatty-acid oxidation and amino-acid metabolism support nucleotide and protein synthesis^14^. These recent discoveries have opened new avenues for possible therapeutic modulation of EC metabolism in cancer and chronic inflammatory disease^15^.

Glycolysis is classically viewed as occurring in the cytosol, with ATP diffusing throughout the cell to reach distant locations such as plasma membrane protrusions and intercellular junctions. ATP diffusion to cell-membrane locations is often limited by the long distances involved and obstruction by the packed cytoskeleton^16,17^. Pioneering studies showed the anchoring of glycolytic enzymes to the erythrocyte plasma membrane^18^, and more recent evidence supports the subcellular compartmentalization of glycolytic and ATP-producing enzymes in other cell types^19^. Examples of this type of local ATP production include the regulation of vesicle traffic along microtubules in neurons by GAPDH^20^, actin cytoskeleton reorganization at lamellipodia by PFKFB3^11^, and the establishment and stability of endothelial cell-cell contacts by the nucleoside-diphosphate kinase (NDPK) NM23^6^. These findings indicate that compartmentalization of ATP-producing enzymes is a general mechanism governing spatio-temporal cellular events^21^. How this ATP compartmentalization occurs is, however, incompletely understood.

During glycolysis, the enzyme pyruvate kinase (PK) is in charge of catalyzing the last step, which converts phosphoenolpyruvate to pyruvate with the generation of one molecule of ATP. PK has three forms: L (liver), R (erythrocytes), and M (muscle). The M form is expressed in most human organs and has two isoforms, M1 and M2, regulated by alternative splicing of the PKM gene^22^. The M1 and M2 isoforms differ in the regulation of their catalytic activity, with PKM1 constitutively active and PKM2 subject to regulation^22–25^. PKM2 function has mainly been analyzed in cancer and inflammation^26,27^. Although PKM2 was considered a master regulator of proliferation, particularly in tumor cells, recent studies have established that the primary cause of cell-cycle arrest in PKM2-null mice or PKM2-depleted cells is compensatory overexpression of PKM1^27,28^. Protein kinase actions have also been linked to PKM2, although the direct contribution of PKM2 and the importance of this activity remain unresolved^29,30^. In the context of angiogenesis, soluble PKM2 dimers released by tumor cells have been reported to increase endothelial cell-tube formation by increasing cell proliferation, migration, and extracellular matrix adhesion; however, the mechanisms of these PKM2 extracellular actions remain unclear^31^. More recently, JMDM8 was shown to regulate human EC sprouting and metabolism through its association with PKM2, but the impact of JMDM8 silencing was only analyzed in the sprout spheroid assay, and no mechanisms were defined^32^. In this study, we explore PKM2 actions in sprouting angiogenesis *in vitro* and *in vivo* and decipher the role of PKM2 subcellular compartmentalization in this process.

## Results

### PKM2 is required for sprouting angiogenesis *in vitro* and *in vivo*

**Figure 1:**
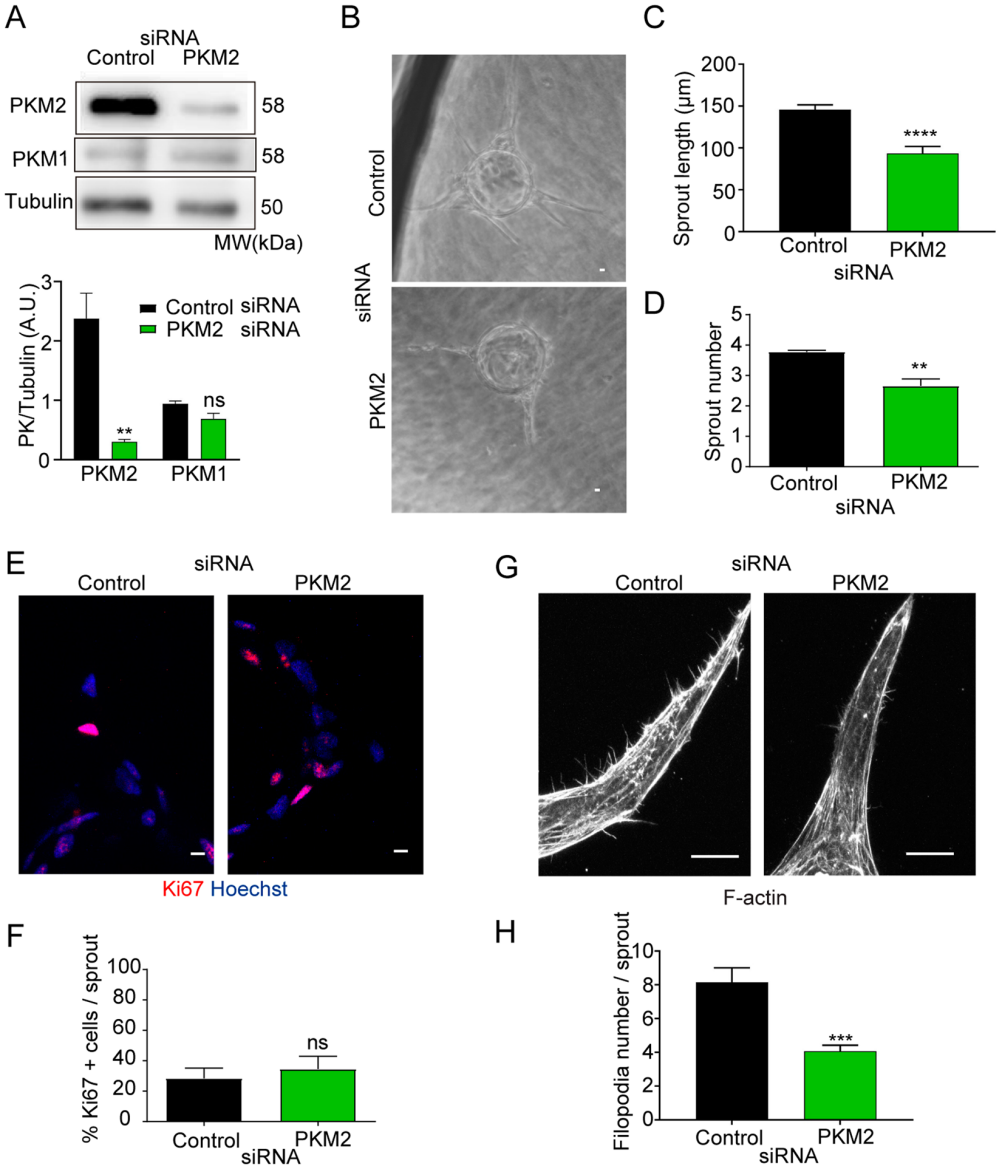
PKM2 is required for *in vitro* endothelial cell sprouting. (**A**) Western blot of PKM2 and PKM1 expression 72 hours after siRNA silencing in HUVECs and quantification versus tubulin included as a loading control; means ± SEM, n = 3, ns non-significant, **p < 0.01 by unpaired Student t-test. (**B**) Bright-field microscopy images of spheroids coated with HUVECs transfected with control or PKM2 siRNA and embedded in fibrin gels for 7 days. Scale bar, 10 µm. (**C**) Sprout length in 3D spheroids; means ± SEM, n = 103 and 38 spheroids formed by control and PKM2 siRNA-silenced cells from one representative experiment of five performed, ****p < 0.0001 by unpaired Student t-test. (**D**) Sprout numbers in 3D spheroids; means ± SEM, n = 27 and 14 spheroids formed by control and PKM2 siRNA-silenced cells from one experiment representative of five performed, **p < 0.01 by unpaired Student t-test. (**E**) Immunofluorescence of Ki67 (red, proliferation) and Hoechst (blue, nuclei) in 3D spheroid sprouts. Scale bar, 10 µm. (**F**) Percentage of Ki67-positive cells per sprout in 3D spheroids; means ± SEM, n = 3 independent experiments, ns non-significant by paired Student t-test. (**G**) Immunofluorescence of F-actin in 3D spheroid sprouts. Scale bar, 10 µm. (**H**) Filopodia number in 3D spheroids; means ± SEM, n = 13 and 15 filopodia in sprouts formed by control and PKM2 siRNA-silenced cells from one representative experiment of five performed, ***p < 0.0001 by Welch´s test. MW, molecular weight. See also Figure S1.

To characterize the role of PKM2 in ECs during sprouting angiogenesis, we specifically silenced PKM2 expression in human umbilical vein ECs (HUVEC) with an isoform-specific siRNA that decreased PKM2 expression without affecting PKM1 levels (Fig. 1A). In the spheroid culture system used to study sprouting angiogenesis, suppression of PKM2 expression was maintained up to the final time point (day 7) (Supplementary Figure S1A,B). Although sprouts developed a proper lumen in both groups (Supplementary Figure S1C), PKM2-silenced EC spheroids produced fewer and shorter sprouts than spheroids formed by control siRNA-transfected ECs (Fig. 1B–D). Silencing PKM2 also resulted in reduced number and shorter endothelial tubes in the ‘hanging drop’ assay, which runs for 24 hours (Supplementary Figure S1D,E). Moreover, sprouts formed by control and PKM2-silenced EC spheroids contained similar proportions of Ki67+ proliferating cells, and tip cells in these spheroids produced fewer filopodia (Fig. 1E–H). Silencing PKM2 with an independent siRNA in mouse lung ECs also impaired sprout formation in the spheroids confirming the need of PKM2 for *in vitro* sprouting angiogenesis (Supplementary Figure S1F,G).

**Figure 2:**
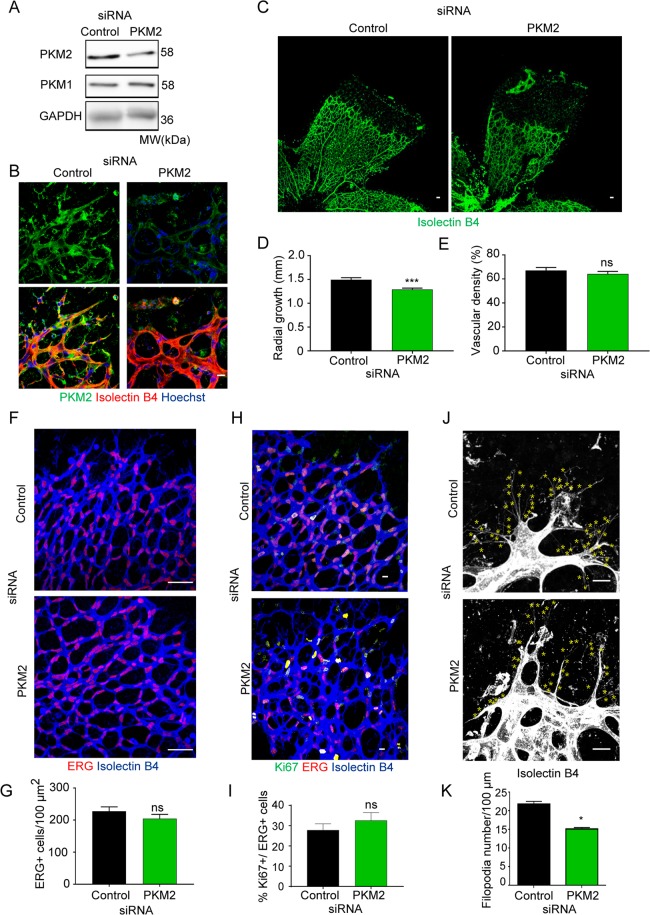
PKM2 silencing results in reduced vascular growth and filopodia number in the postnatal mouse retina. (**A**) Western blot of PKM2 and PKM1 expression in protein extracts from mouse retinas obtained 72 hours after intravitreal siRNA-injection. GAPDH is included as a loading control; n = 3 mice per condition. (**B**) Immunofluorescence of isolectin B4 (red, vessels), PKM2 (green), and nuclei (blue, Hoechst) in whole-mount P6 mouse retinas 72 hours after intravitreal siRNA-injection. Scale bar, 10 µm. (**C**) Immunofluorescence of isolectin B4 (green, vessels) in whole-mount P6 mouse retinas 72 hours after intravitreal siRNA-injection. Scale bar, 50 µm. Disconnected Erg/IB4-positive structures correspond to rests of hyaline membrane fragments. (**D**) Radial vascular growth in mouse retinas (P6) 72 hours after intravitreal siRNA-injection, means ± SEM, n = 8 mice per condition, ***p < 0.001 by Mann-Whitney test. (**E**) Vascular density in mouse retinas (P6) 72 hours after intravitreal siRNA-injection, means ± SEM, n = 4 mice per condition, ns non-significant by unpaired Student t-test. (**F**) Immunofluorescence ERG (white, endothelial cell nuclei) in whole-mount P6 mouse retinas 72 hours after intravitreal siRNA-injection. Scale bar, 50 µm. (**G**) ERG positive cells per vessel area in P6 mouse retinas 72 hours after intravitreal siRNA-injection, means ± SEM, n = 4 mice per condition, ns non-significant by unpaired Student t-test. (**H**) Immunofluorescence of isolectin B4 (blue, vessels), Ki67 (green, proliferation), and ERG (red, endothelial cell nuclei) in whole-mount P6 mouse retinas 72 hours after intravitreal siRNA-injection. Scale bar, 10 µm. (**I**) Percentage of Ki67-positive cells per total ERG-positive cells in P6 mouse retinas 72 hours after intravitreal siRNA-injection; means ± SEM, n = 4 mice per condition, ns non-significant by unpaired Student t-test. (**J**) Immunofluorescence of isolectin B4 (white, vessels) in whole-mount P6 mouse retinas 72 hours after intravitreal siRNA-injection. Each yellow asterisk marks one filopodia. Scale bar, 10 µm. (**K**) Number of filopodia per 100 μm of vascular front in P6 mouse retinas 72 hours after intravitreal siRNA-injection; means ± SEM, n = 4 mice per condition, *p < 0.05 by unpaired Student t-test. MW, molecular weight.

To determine whether PKM2 was also required for sprouting angiogenesis *in vivo*, P6 mice were given an intravitreal injection of PKM2 siRNA, and we examined the retinal vasculature after 72 h. Western blot of retina extracts showed reduced PKM2 protein expression, and immunofluorescence analysis confirmed decreased PKM2 in the vasculature (Fig. 2A,B). PKM2-silencing in retina diminished radial vascular growth with no impact on vascular density (Fig. 2C–E). This prompted us to analyze EC number and proliferation. PKM2-silenced and control-siRNA treated retinas had similar densities of ERG+ cells and the same proportion of Ki67+ ECs (Fig. 2F–I). Matching the *in vitro* analysis, filopodia number was lower in PKM2-silenced retinas (Fig. 2J,K). Together, these observations show that PKM2 is required for sprouting angiogenesis *in vitro* and *in vivo* by mechanisms that do not seem to involve EC proliferation.

### PKM2 is located at VE-cadherin-expressing endothelial cell junctions

**Figure 3:**
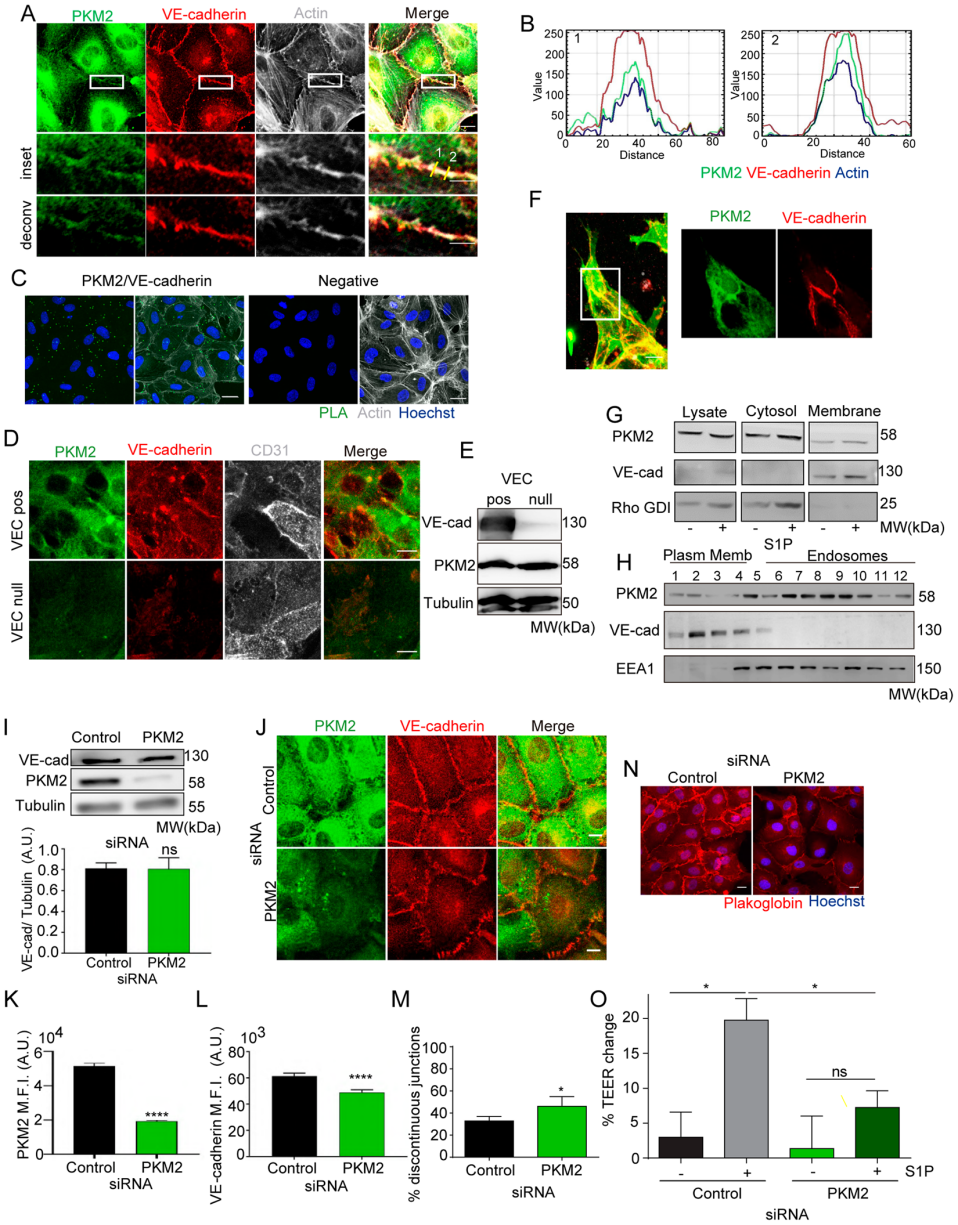
PKM2 is localized at VE-cadherin junction compartment. (**A**) Immunofluorescence of PKM2 (green), VE-cadherin (red) and F-actin (grey) in confluent HUVECs; single color and merged images are shown. Magnified views and de-convolved images of boxed areas below. Scale bars, 10 µm and 5 µm in the magnified views. (**B**) RGB (red, green, blue) profile of PKM2 (green), VE-cadherin (red) and F-actin (blue) intensity at areas of the endothelial cell junction marked by the yellow lines in A. (**C**) Representative merged images of Hoechst (blue) and proximity ligation assay (PLA, green) for PKM2 and VE-cadherin (left) and together with F-actin (grey; right) in HUVECs. PLA of rabbit and goat IgGs is also shown (negative). Scale bar, 10 µm. (**D**) Immunofluorescence of PKM2 (green), VE-cadherin (red) and CD31 (grey) in confluent VE-cadherin-expressing (VEC-pos) and VE-cadherin–deficient (VEC-null) mouse endothelial cells; single color and merged images of green and red channels are shown. Scale bar, 10 µm. (**E**) Western blot of VE-cadherin and PKM2 in lysates from VEC-pos and VEC-null endothelial cells. Tubulin is included as a loading control. MW, molecular weight. (**F**) Immunofluorescence of PKM2 (green), VE-cadherin (red) and F-actin (grey) in 3D spheroid sprouts. Scale bar, 10 µm. (**G**) Western blot of PKM2 in total lysate and in cytosol and membrane fractions of confluent HUVECs treated with S1P or vehicle (left) and transfected with control or PKM2. VE-cadherin and Rho-GDI are included as markers of plasma membrane and cytosol. MW, molecular weight. (**H**) Western blot of PKM2 in membrane subfractions obtained from confluent HUVECs by density gradient centrifugation. VE-cadherin and EEA1 are included as markers of plasma membrane and endosomes. MW, molecular weight. (**I**) Western blot and quantification of VE-cadherin in HUVECs transfected with control or PKM2 siRNA. Tubulin is included as a loading control. Means ± SEM, n = 3 independent experiments, ns non-significant by unpaired Student t-test. (**J**) Immunofluorescence of VE-cadherin (red) and PKM2 (green) in siRNA-silenced confluent HUVECs. Scale bar, 10 µm. (**K** and **L**) Mean fluorescent intensity (M.F.I.) of PKM2 (K) and VE-cadherin (L) at the junctions of siRNA-silenced confluent HUVECs; means ± SEM, n = 5 independent experiments, ****p < 0.0001 by Mann-Whitney test. (**M**) Percentage of discontinuous VE-cadherin-junctions; means ± SEM, n = 3 independent experiments, *p < 0.05 by unpaired Student t-test. (**N**) Immunofluorescence of plakoglobin (red) and nuclei (blue, Hoechst) in siRNA-silenced confluent HUVECs. Scale bar, 10 µm. (**O**) Change in trans-endothelial electrical resistance (TEER) across monolayers of siRNA-silenced HUVECs left untreated or treated with 1 μM S1P for 3 hours; means ± SEM, n = 3 independent experiments, ns non-significant, *p < 0.05 by one-way ANOVA with Sidak post-test. See related Figure S2.

Although PKM2 was mostly found in the cytoplasm of ECs, high-resolution confocal microscopy images, image deconvolution and profile intensity analysis revealed however that a pool of PKM2 localized close to VE-cadherin and cortical F-actin at the junctions (Fig. 3A,B). We confirmed PKM2 vicinity to VE-cadherin at the endothelial junctions by proximity ligation assays (Fig. 3C). Moreover, in spite of similar PKM2 protein levels, PKM2 was barely observed at the junctions of VE-cadherin-null endothelial cells compared to VE-cadherin-positive ones (Fig. 3D,E). PKM2 was also found nearby the junctions of ECs stimulated with sphingosine-1-phosphate (S1P), an inducer of EC junction remodeling and reinforcement^33^ (Supplementary Figure S2A), and of 3D EC sprouts *in vitro* (Fig. 3F), without significant differences in PKM2 presence at the junctions of tip and stalk cells (PKM2 MFI_tip_ = 144.4 ± 22.82 and MFI_stalk_ = 156.3 ± 20.3, n = 19 sprouts). PKM2 was also detected at F-actin-rich filopodia and lamellipodia of migrating ECs in 3D and 2D respectively (Supplementary Figure S2B,C), in line with the reported PKM2/F-actin association^34^.

In accordance with the immunofluorescence data, PKM2 was found by western blot in both cytosolic and membrane EC fractions and the specificity of this compartmentation was shown by reduced PKM2 abundance in the membrane fraction of PKM2-silenced ECs (Fig. 3G). Cell membrane subfractionation further revealed that PKM2 was present in low-density fractions containing plasma membrane and in endosomes (Fig. 3H).

### PKM2-silencing destabilizes junctions and impairs EC collective migration

Productive angiogenesis depends on VE-cadherin-mediated EC junction stability and dynamics^35,36^. We therefore investigated the influence of PKM2 on EC-junctional VE-cadherin at baseline and upon junction remodeling induced by S1P. In spite of no change in total VE-cadherin protein expression, PKM2-silenced ECs showed lower cell-junction VE-cadherin intensity than control ECs, together with a higher proportion of discontinuous junctions at baseline and after S1P treatment (Fig. 3I–M and Supplementary Figure S2D–F). Junctions formed by PKM2-silenced ECs also showed a clear decrease in plakoglobin, a marker of stable VE-cadherin cell-cell contacts (Fig. 3N and Supplementary Figure S2G). Indeed, PKM2-silencing with an independent siRNA confirmed a similar altered junctional VE-cadherin pattern in mouse lung ECs (Supplementary Figure 2H–J). Finally, PKM2-silenced cells showed a weakened response to S1P-driven reinforcement of the EC barrier, as indicated by lower transendothelial electrical resistance (TEER) values (Fig. 3O). These data indicate defects in VE-cadherin–mediated EC barrier in the absence of PKM2 in remodeling conditions.

**Figure 4:**
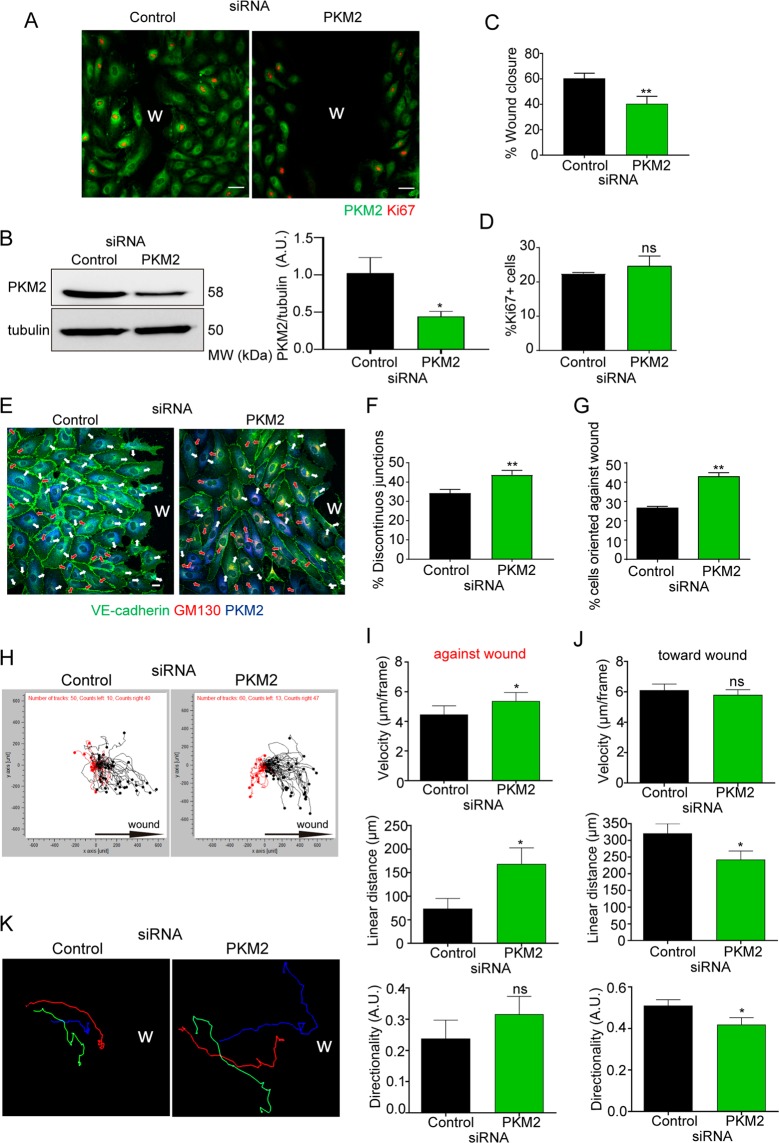
PKM2 silencing leads to misorientation and impaired collective migration of ECs. (**A**) Immunofluorescence of PKM2 (green) and Ki67 (red, proliferation) in siRNA-silenced HUVECs 16 hours after scratch wounding. Scale bar, 50 µm. W, wound. (**B**) Western blot and quantification of PKM2 expression in silenced-HUVEC cells at the end of the scratch assay; means ± SEM, n = 3 independent experiments, *p < 0.05 by Mann-Whitney test. MW, molecular weight (**C** and **D**) Percentage of wound closure (**C**) and of Ki67-positive cells (**D**) 16 hours after scratch wounding; means ± SEM, n = 3 independent experiments, ns non-significant and **p < 0.01 by unpaired Student t-test. (**E**) Immunofluorescence of VE-cadherin (green), PKM2 (blue), and GM130 (red) in siRNA-silenced HUVECs 16 hours after scratch wounding. W, wound. White and red arrows indicate cell polarization toward and away from the wound, respectively; Scale bar, 10 µm. (**F** and **G**) Percentage of discontinuous junctions (**F**) and of endothelial cells oriented against the wound (**G**) in siRNA-silenced HUVECs 16 hours after scratch wounding; means ± SEM, n = 3 independent experiments, **p < 0.01 by unpaired Student t-test. (**H**) Single-cell track analysis of siRNA-silenced HUVECs in the scratch assay; n = 3 independent experiments. Black and red tracks indicate cells migrating toward and against the wound. (**I** and **J**) Velocity, linear (Euclidean) distance and directionality of single cells moving against (**I**) and toward the wound (**J**); means ± SEM, n = 50–60 cells from 3 independent experiments, ns non-significant and *p < 0.05 by Mann-Whitney test. (**K**) Representative single-cell tracks of three adjacent siRNA-silenced ECs migrating during the scratch assay. See related Figure S3 and Movies S1 and S2.

A recognized function of VE-cadherin at cell-cell junctions is to conduct polarity signals while maintaining barrier integrity during collective migration^36^. In line with the observed junctional defects, PKM2-silenced HUVECs were significantly less efficient than control siRNA-transfected ECs at closing the wound area in the scratch assay without showing differences in cell proliferation (Fig. 4A–D). Accordingly, inhibition of proliferation did not change the defective wound closure and PKM2 silencing did not affect EC basal growth and proliferation (Supplementary Figure S3A–D). We next assessed the role of PKM2 in the orientation and directionality of migrating EC cells by quantifying Golgi apparatus polarization and by time-lapse microscopy^37,38^. PKM2-silenced migrating EC cultures showed more discontinuous junctions and this correlated with a higher proportion of cells polarized against the wound than control cultures (Fig. 4E–G). Time-lapse recording and single-cell track analysis confirmed that PKM2-silenced ECs migrated faster and for longer linear (Euclidean) distances opposite to the wound but for shorter distances and less directional toward the wound compared with controls (Fig. 4H–J and Movies S1–S2). Moreover, PKM2 silencing impaired collective migration of adjacent endothelial cells (Fig. 4K).

### PKM2 is required for endothelial cell junction remodeling during angiogenic sprouting *in vitro* and *in vivo*

**Figure 5:**
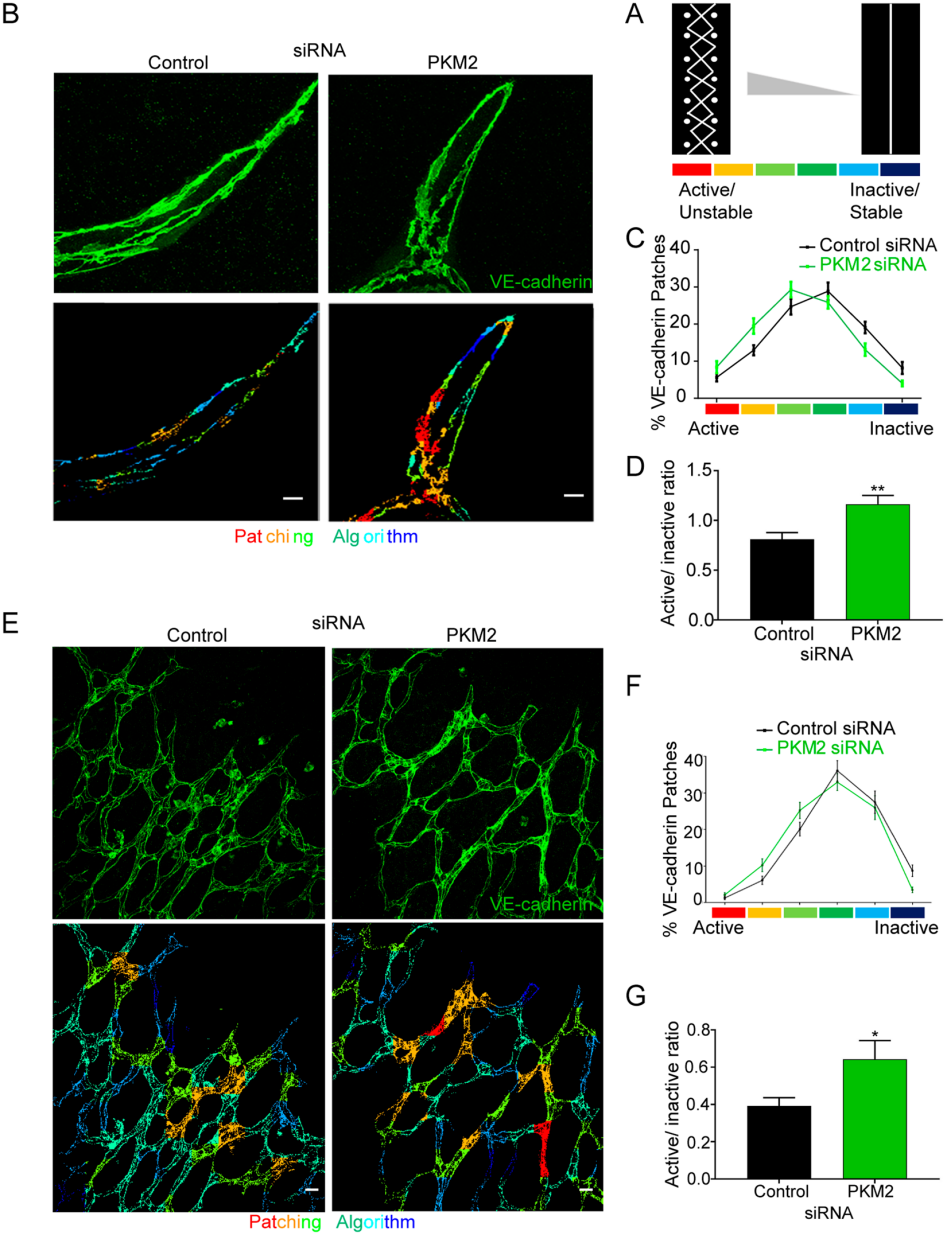
PKM2 is required for endothelial-cell junction remodeling during sprouting angiogenesis *in vitro* and *in vivo*. (**A**) Scheme illustrating the patching algorithm classification of VE-cadherin active (unstable)/inactive (stable) junctions adapted from Bentley *et al*., 2014. (**B**) Immunofluorescence of VE-cadherin (green, top) in 3D spheroid sprouts. Pseudo-colored images according to the patching algorithm classification are shown below. Scale bar, 10 µm. (**C** and **D**) Percentage of VE-cadherin active and inactive junction patches (**C**) and their ratio (**D**) in 3D spheroid formed by siRNA-silenced HUVECs; means ± SEM, n = 3 independent experiments. **p < 0.01 by Mann-Whitney test. (**E**) Immunofluorescence of VE-cadherin (green, top) in P6 mouse whole-mount retinas 72 hours after intraocular siRNA injection. Pseudo-colored images according to the patching algorithm classification are shown below. Scale bar, 10 µm. (**F** and **G**) Percentage of VE-cadherin active and inactive junction patches (**F**) and their ratio (**G**) in P6 mouse retinas 72 hours after siRNA injection; means ± SEM, n = 4 mice, *p < 0.05 by Welch’s test.

In addition to maintenance of junction stability and to collective migration in 2D, VE-cadherin dynamics are also essential for functional EC arrangements during 3D sprouting angiogenesis, and this manifests as a balanced distribution of stable and unstable EC junctions^35^. To determine the role of PKM2 in regulating this mosaicism, we quantified the distribution of EC junction patches by classifying the morphology of EC junctions based on their VE-cadherin pattern, from discontinuous (active/unstable) to continuous (inactive/stable) according to the reported ‘patching algorithm’^35^. The 3D sprouts formed *in vitro* by PKM2-silenced human ECs showed an altered EC-junction patch distribution, with a higher abundance of patches containing active/unstable junctions than 3D sprouts formed by control siRNA-transfected ECs; this was reflected by a change in the active/unstable:inactive/stable patch ratio (Fig. 5A–D). This phenotype was also confirmed *in vivo* in the vasculature of PKM2-silenced postnatal retinas (Fig. 5E–G).

### PKM2 activity is involved in the regulation of EC junctions and sprouting angiogenesis

In addition to its action in glycolysis, PKM2 has been linked to non-glycolytic functions^29,30^. To determine whether the effects of PKM2 absence on EC junctions and sprouting angiogenesis are related to pyruvate kinase activity, we treated ECs with shikonin, a specific inhibitor of the PKM2 isoform^39^. Pharmacological inhibition of PKM2 activity increased the number of discontinuous junctions in basal and S1P-treated human ECs (Supplementary Figure S4A,B) and recapitulated the rest of effects of PKM2-silencing. Shikonin reduced the number and length of sprouts in the 3D spheroid assay and impaired wound closure and EC collective migration without influencing EC proliferation (Supplementary Figure S4C–G and Movies S3-S4). In contrast, the general glycolysis inhibitor 2-deoxyglucose (2-DG), in addition to impairing junction stability and EC migration, also decreased EC proliferation (Supplementary Figure S4H–K). PKM2 activity was also required *in vivo* since intravitreal injection of shikonin reduced vascular radial growth and filopodia number in P6 mice while having no effect on vascular density, EC number, or the percentage of proliferating ECs, thus reproducing the phenotype observed in the vasculature of PKM2-silenced retinas (Supplementary Figure S5A–I). Intravitreal shikonin injection also shifted the active/unstable:inactive/stable junction ratio by increasing the proportion of active junctions in the P6 retinal vasculature (Supplementary Figure S5J–L).

### PKM2-silencing decreases ATP nearby EC junctions

**Figure 6:**
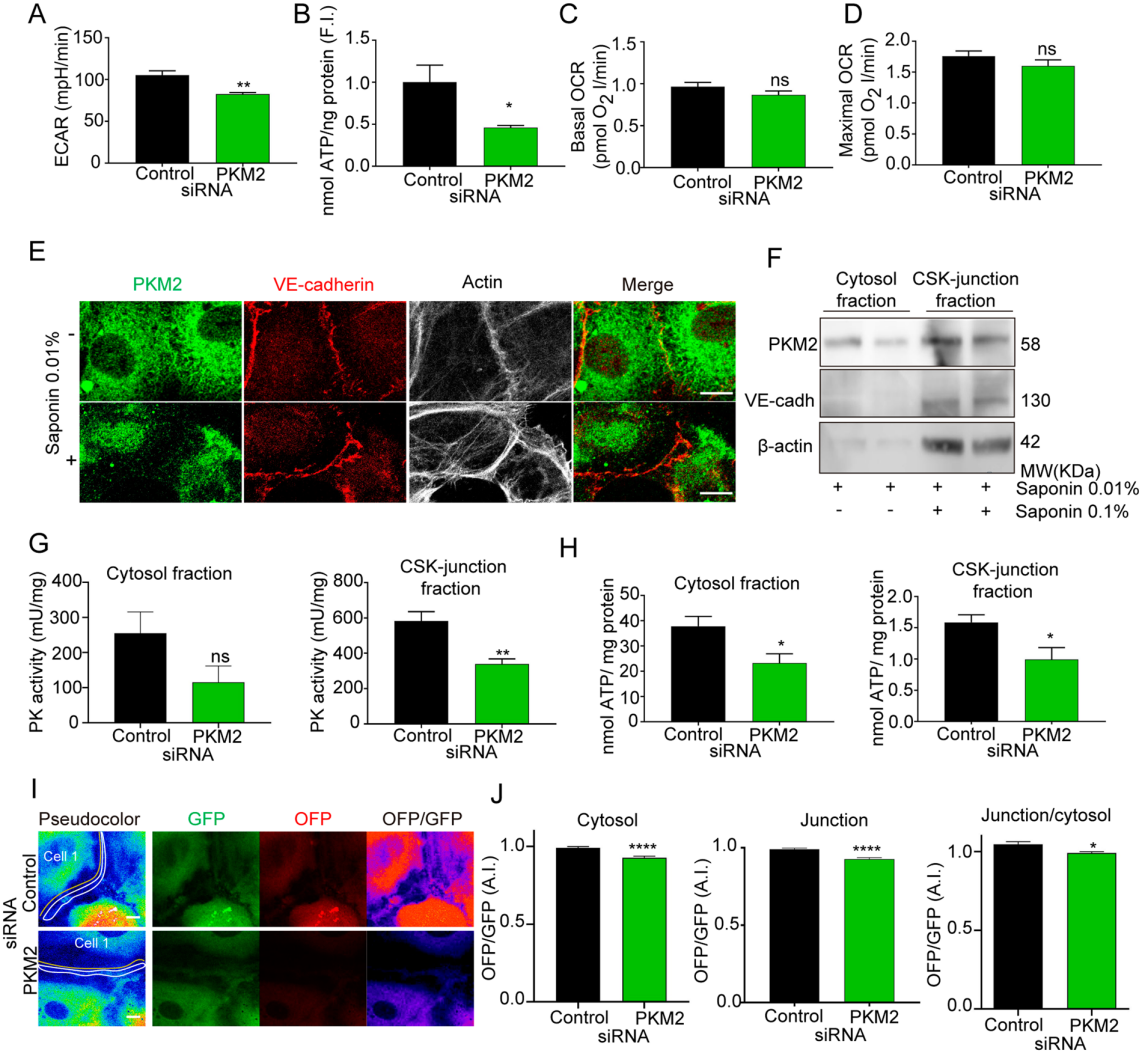
PKM2 activity is required for ATP levels at EC junctions. (**A**) Extracellular acidification ratio (ECAR) in HUVECs transfected with control or PKM2 siRNA; means ± SEM, n = 3 independent experiments.**p < 0.01 by Mann-Whitney t-test. (**B**) Total ATP per ng protein in HUVECs transfected with control or PKM2 siRNA, quantified as fold induction (F.I.) versus control; means ± SEM, n = 8 independent samples analyzed in 2 sets of experiments. *p < 0.05 by Mann-Whitney t-test. (**C** and **D**) Baseline (**C**) and maximum (**D**) oxygen consumption ratio (OCR) in HUVECs transfected with control or PKM2 siRNA; means ± SEM, n = 3 independent experiments, ns non-significant by Mann-Whitney t-test. (**E**) Immunofluorescence of PKM2 (green), VE-cadherin (red) and F-actin (grey) in HUVECs permeabilized or not with 0.01% saponin. Merged image of green and red channels is also shown. Scale bar, 10 µm. (**F**) Western blot of PKM2 in fractions extracted from HUVECs with 0.01% saponin (cytosol enriched) or 0.01% + 0.1% saponin (cytoskeleton (CSK)-junction enriched). VE-cadherin and β-actin are included as markers of cell junctions and cytoskeleton. MW, molecular weight. (**G** and **H**) Pyruvate kinase activity (**G**) and ATP amount (**H**) normalized to protein content in fractions extracted as in F from HUVECs transfected with control or PKM2 siRNA; means ± SEM, n = 8 independent samples analyzed in 2 sets of experiments, ns non-significant, *p < 0.05 and **p < 0.001 by unpaired Student t-test. (**I**) Images of the GFP, OFP and OFP/GFP channels from live microscopy of GO-ATeam1-transduced HUVECs silenced with control or PKM2 siRNA. The yellow and white lines mark in the GFP channel the cytosol and EC junction areas for OFP/GFP quantification. Scale bar, 10 µm. (**J**) OFP/GFP intensity ratio at cytosol, EC junctions and EC junction/cytosol of GO-ATeam1-transduced HUVECs transfected with control or PKM2 siRNA as fold induction respect control siRNA cells in each experiment, means ± SEM, n = 100–109 cells from 5 independent experiments analyzed per condition, ****p < 0.0001 by unpaired Student t-test. See related Figures S4, S5 and S6.

Given the requirement of PKM2 kinase activity for its actions on EC junctions and sprouting angiogenesis, we next assessed the impact of PKM2 on EC ATP production. Analysis of the global metabolic state revealed that PKM2-silenced ECs had a lower glycolysis flux than control siRNA-transfected cells, revealed by a relative ~20% drop in the extracellular acidification ratio (ECAR) (Fig. 6A). This was reflected in significantly lower total ATP production (Fig. 6B). Mitochondrial activity remained intact, with similar basal and maximal oxygen consumption ratios (OCR) in PKM2 and control siRNA-silenced human ECs (Fig. 6C,D).

Following the protocol reported for other glycolytic enzymes^16,20^, we next performed sequential cell lysis with increasing proportions of saponin to allow the extraction of two fractions, one enriched for cytosol and the other less soluble for cytoskeleton and EC junctions (CSK-junction enriched) (Fig. 6E,F). Pyruvate kinase activity was ~1.8-fold higher in the CSK-junction enriched sub-fraction than in the cytosol; specificity for PKM2 was confirmed by decreased PK activity in both compartments when PKM2 expression was silenced (Fig. 6G).

To assess whether PKM2-silencing impaired local ATP levels, particularly at CSK-junction compartment, we quantitated ATP in saponin-extracted sub-fractions. PKM2-silencing in mouse and human ECs significantly decreased ATP production in the cytosol, by ~50%, and the CSK-junction enriched sub-fraction, by ~40% (Supplementary Figure S6A and Fig. 6H), in accordance with the reduced PK activity (Fig. 6G). To ensure quantification of ATP levels nearby EC junctions, we transduced HUVECs with lentivirus encoding the FRET-based ATP sensor GO-ATeam1^40^. PKM2 silencing resulted in a significant reduction in the GO-ATeam1 FRET signal at both cytosol and junctions (as in subcellular fraction analysis), but the ratio of ATP levels at junctions versus cytosol was significantly decreased indicating a major impact of PKM2 absence on ATP levels at the junctional region of 2D human EC monolayers (Fig. 6I,J). We observed similar results in GO-ATeam1-transduced HUVEC when PKM2 activity was inhibited by shikonin in line with the reduction of ATP levels in the CSK-junction fraction (Supplementary Figure S6B,C). These findings indicate that PKM2-silencing reduces ATP levels nearby EC junctions.

### PKM2 and local ATP regulate VE-cadherin dynamics and internalization at EC junctions

We next explored whether reduced ATP production in the CSK-junction compartment in PKM2 absence/inhibition may affect VE-cadherin turnover^41^. Gradient sub-fractionation experiments revealed that PKM2 inhibition by shikonin increased the proportion of VE-cadherin present at the plasma membrane versus endosomes compared to control ECs (Supplementary Figure S6D).

**Figure 7:**
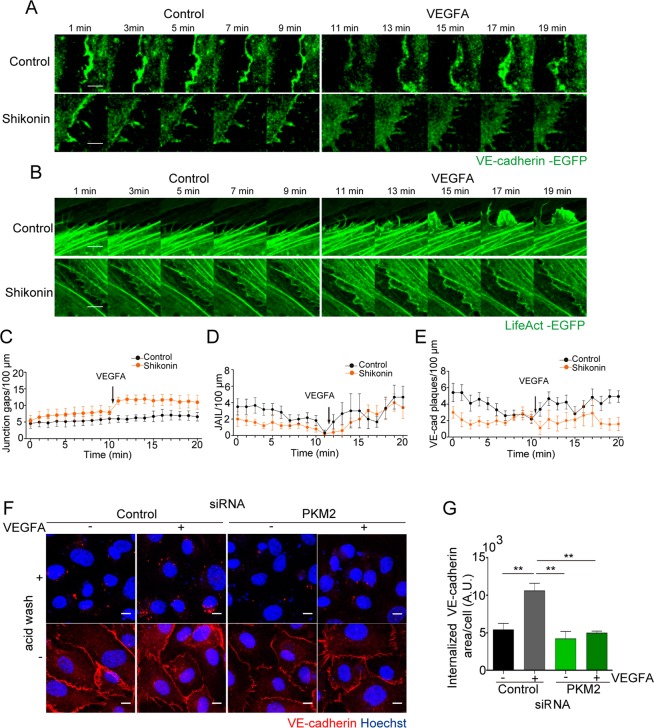
PKM2 is required for junction dynamics and VE-cadherin internalization. (**A** and **B**) Still images from live microscopy of HUVECs transduced with lentivirus coding for VE-cadherin-EGFP (**A**) or LifeAct-EGFP (**B**) for 10 min prior and after addition of 20 ng/ml of VEGFA; untreated and shikonin-treated cells are shown. Scale bar, 5 µm. (**C**) Number of inter-cellular gaps at junctions of VE-cadherin-transduced HUVEC along time. (**D** and **E**) Number of JAIL and of VE-cadherin plaques at the lateral junctions of LifeAct-EGFP and VE-cadherin-EGFP-transduced HUVECs, respectively; means ± SEM, n = 6 junctions for 2 independent experiments. (**F**) Immunofluorescence of VE-cadherin (red) and Hoechst (blue, nuclei) before or after acid wash in HUVECs transfected with control or PKM2 siRNA and treated with 20 ng/ml VEGFA for 15 minutes. Scale bar, 10 µm. (**G**) Internalized VE-cadherin positive area per number of cells in arbitrary units (A.U.); n = 3 independent experiments, **p < 0.01 by one-way ANOVA with Sidak post-test. See related Figure S6 and Movies S5–S8 and S11–S14.

Live imaging of HUVEC transduced with lentivirus coding for VE-cadherin-EGFP or LifeAct-EGFP in basal conditions and after stimulation with VEGFA allowed us to analyze VE-cadherin, and F-actin dynamics at EC junctions^42^. Time-lapse microscopy of VE-cadherin-EGFP-expressing HUVEC showed that PKM2 inhibition by shikonin led to increased numbers of inter-cellular gaps mostly after VEGFA treatment compared to control ECs (Fig. 7A,C; Movies S5–S8); similar gaps were observed in VE-cadherin-EGFP-transduced HUVEC silenced for PKM2 (Supp Figure S6E and Movies S9–S10). These data confirmed the enhanced junction instability observed in 2D endothelial monolayers in static conditions (Fig. 3H–M). In LifeAct-EGFP-expressing ECs, we visualized small active F-actin protrusions in the lateral border preceding VE-cadherin ‘plaques’ and corresponding to ‘junction-associated intermittent lamellipodia’ (JAIL), which increased in size and persistence after VEGFA stimulation in control cells as reported^42^ (Movies S11–S14). Notably, PKM2 inhibition by shikonin significantly reduced the number of JAIL and VE-cadherin plaques in untreated and VEGFA-stimulated cells compared to controls (Fig. 7A,B,D,E).

We next investigated directly the impact of PKM2 inhibition on VE-cadherin internalization in basal conditions and after stimulation with VEGFA. VEGFA-triggered VE-cadherin internalization is a classic clathrin-dependent/ATP-consuming event^43,44^. Notably, PKM2 silencing in ECs led to a significant reduction of VEGFA-induced VE-cadherin internalization compared to controls (Fig. 7F,G), arguing for decreased local ATP level as a mediator of such effect. We obtained a similar phenotype in human ECs treated with shikonin (Supplementary Figure S6F).

Our data support a model in which PKM2 absence/inhibition reduces ATP levels nearby endothelial junctions thus hindering VE-cadherin clathrin-mediated internalization and JAlL formation and overall resulting in impaired junction remodeling, collective migration, and angiogenic sprouting.

## Discussion

This study presents the first description of the impact of subcellular compartmentalization of PKM2 on ATP levels, VE-cadherin internalization and EC junction rearrangements, and sprouting angiogenesis *in vitro* and *in vivo*.

PKM2 expression has been correlated with cell proliferation, particularly in tumor cells^26^. Our data establish that PKM2 is not required for EC proliferation, since PKM2-silenced and shikonin-treated ECs showed no differences from controls *in vitro* or *in vivo*. Despite the proposed role of PKM2 in cancer-cell proliferation, recent findings from Vander Heiden’s group and others indicate that cell-cycle arrest in the absence of PKM2 is likely driven by compensatory PKM1 upregulation^27^. In this study, PKM2-null mice displayed distinct patterns of tumor-cell proliferation depending on PKM1 expression, such that if PKM1 was expressed, proliferation capacity was reduced^27^. Moreover, PKM2-deficient primary cells derived from this mouse model also showed compensatory PKM1 upregulation, leading to impaired cell proliferation^28^. Corroborating this, proliferating embryo regions which would normally express PKM2 have been shown to initiate PKM1 expression in PKM2-null embryos, which is accompanied by a slowing of cell division and unexpected normal embryonic development^45^. In our experiments, PKM1 expression did not increase in PKM2-silenced ECs, possibly explaining the lack of any impact on EC proliferation. This is in contrast with recent reports claiming that PKM2 knockout reduced EC proliferation in which the compensatory increase of PKM1 might be a confounding factor to interpret direct PKM2 effects in EC proliferation^46,47^. Our findings further support the functional segregation of metabolic enzymes in regulating the spectrum of EC responses required for angiogenesis^14^.

PKM2 has been reported to both favor and decrease cancer cell migration^48–50^. Our study is in line with recent reports on the requirement of PKM2 for endothelial cell migration^46,47^. Other glycolytic enzymes have been shown to positively regulate cell migration, for example PGK1 in tumor cells^51^, PFKFB3 in ECs^11^, and HK2 in lymphatic ECs^13^. Our findings show that PKM2 activity is required for EC collective migration, evidenced by impaired EC orientation and migration in the scratch assay and by the reduced 3D sprout-length and retinal vascular radial outgrowth without changes in proliferation.

Our findings also provide insight into how differential VE-cadherin dynamics and mosaic junction activity influence productive angiogenesis. PKM2 loss reduced junctional VE-cadherin and increased the proportion of active/unstable junctions, thus compromising the balance between inactive/stable and active/unstable junctions and destabilizing the endothelial barrier. These findings thus indicate that PKM2 is primarily required for proper VE-cadherin–mediated endothelial cell-junction stability. The ‘patching algorithm’ developed by Bentley^35^ has been especially useful in gaining insight into this process because it gives not only absolute numbers of stable and unstable junctions but also their clustered distribution. Recent studies into the role of EC junctions in angiogenesis provide evidence that they not only constitute a barrier and a signaling platform but also fine-tune cellular processes of paramount importance for efficient directionality and expansion of capillary sprouts. For example, VE-cadherin fingers from the pulling EC are essential for collective and cohesive EC migration during sprout extension^36^; we confirm that defective VE-cadherin at junctions by PKM2 absence or inhibition results in impaired collective migration. Our findings also demonstrate that VE-cadherin internalization which is required for EC junction stability and remodeling, depends on PKM2 activity, likely through the provision of ATP for vesicle movement^20^ since VEFGA-induced VE-cadherin endocytosis is clathrin-dependent and thus ATP-consuming^52^. PKM2-generated ATP at junctions could additionally favor actomyosin contractility or the phosphorylation of enzymes implicated in cell-cell junction stabilization or remodeling^6,53,54^.

PKM2 silencing also led to reduced numbers of filopodia in the endothelial tip cells. Since filopodia positively regulate cell motile velocity, they ultimately affect sprouting^55,56^. This phenotype could be related to the reported association of PKM2 with F-actin^34^ since although our live imaging data favor the hypothesis of a primary impact of PKM2 inhibition on VE-cadherin internalization and JAIL formation, we cannot rule out an additional action on F-actin dynamics. Thus, PKM2 could provide ATP near to dynamic membrane protrusions such as filopodia and lamellipodia, and together with other enzymes such as PKFB3 form the actin-bound ‘glycolytic hub’ proposed by Carmeliet’s group^19,57^. Whether a similar complex also forms at EC junctions deserves however further analysis.

VE-cadherin is the master regulator of EC junction formation and stability, but recently new players have been identified. For example, vinculin restrains junctions during remodeling and pacsin2 inhibits asymmetric VE-cadherin internalization, protecting EC-junction integrity from mechanical forces; moreover, the YAP/TAZ pathway increases VE-cadherin turnover, promoting cell migration while maintaining barrier function^58–60^. EC junctions, and particularly VE-cadherin, are thus dynamically and exquisitely remodeled during angiogenesis. Nevertheless, in spite of the recognized importance of metabolic pathways in EC responses, very little is known about how metabolism impacts EC junction stability and dynamics. Only the glycolytic enzyme PFKBF3 has been shown to modulate EC junctions, by an as-yet unknown mechanism^61^. Although PKM2 loss has been claimed to alter EC junction stability by non-enzymatic actions^46,47^, to our knowledge the present report is the first to demonstrate the presence of a pool of a glycolytic enzyme nearby the junctions of confluent ECs by immunostaining and biochemical approaches. Moreover, we show that PKM2 is required for ATP-dependent processes as VEFGA-induced clathrin-mediated VE-cadherin endocytosis^44^ and thus for junction stability^41^. The fact that PKM2 at the CSK-junction enriched compartment has higher pyruvate kinase activity than cytosolic PKM2 suggests that the activity of the CSK-junction–associated PKM2 is positively regulated by mechanisms that may involve tetramer stabilization upon F-actin binding, similar to what occurs with PFKFB3 at lamellipodia^57^. The activity of PKM2 tetramers at cell-cell junctions may also be regulated by local interaction with allosteric activators or posttranslational modifications^62^. This also raises the question of how PKM2 traffics toward and locates at the plasma membrane. PKM2 interacts with huntingtin^63^, which associates with GAPDH in vesicles traveling along microtubules in neurons^20^, and it has recently been found enriched at these motile vesicles^64^. It is therefore possible that PKM2 also traffics on microtubules alone or together with GAPDH in ECs. Furthermore, we demonstrate that VE-cadherin is required for PKM2 localization at the endothelial junctions. Whether PKM2/VE-cadherin association is direct or mediated by an intermediate protein as β-catenin, a renowned VE-cadherin partner also shown to interact with PKM2 in other contexts^65^, deserves further investigation.

The defective vasculature of PKM2-silenced and shikonin-treated retinas in postnatal mice supports the recent evidences of a requirement for PKM2 in angiogenesis *in vivo*^46,47^. In our context, the vascular impairment affected radial growth and filopodia number without influencing EC proliferation, and the phenotype correlates with the altered ratio of stable and unstable EC junctions. The possible clinical application of these findings to stimulate or inhibit PKM2 activity in disease needs to be carefully considered. In pathological angiogenesis, the proportion of active junctions increases (with a discontinuous pattern)^35^, and sprouting defects are related to impaired endothelial junctions^66,67^. In contrast, there is no evidence that an increased proportion of inactive junctions (with a continuous pattern) has any effect on angiogenesis^68^. PFKFB3, a key glycolysis regulator, has been proposed as a target for inducing normalization of EC junctions^61,69^. In a model of high-dose-VEGFA–induced pathological retinal angiogenesis^35,70^, the PFKFB3 inhibitor 3PO decreased glycolysis by 15% and normalized the vasculature^69^. This effect was also observed in 3PO-treated tumor ECs^61^. However, treatment of ECs with higher 3PO concentrations, inhibiting glycolysis by 40%, induced an increase in discontinuous junctions rather than vessel normalization^71^. This finding underlines the importance of the level of glycolysis inhibition in determining the final angiogenic outcome. In line with this, we observed increased instability of VE-cadherin EC junctions upon PKM2-silencing, which also decreases glycolysis by 40%, and with the global glycolysis inhibitor 2-DG, which almost abolishes glycolysis. Moreover, high concentrations of exogenous ATP also increase discontinuous junctions and decrease TEER in brain endothelial cells^72^. Together, these observations suggest a dual effect of ATP, with a precisely adjusted level required for proper dynamic EC junction rearrangement. Metabolic regulation of EC junctions thus appears to be a complex process that depends on the quantity of ATP produced, the step in the glycolytic pathway involved, and where the implicated enzyme is located. Given the likelihood that glycolysis inhibition will have a ‘dose response’ effect on angiogenesis, studies are warranted to address the impact of PKM2 silencing or inhibition on pathological angiogenesis.

Our findings show that PKM2 makes an essential contribution to angiogenesis *in vitro* and *in vivo* by providing ATP for the regulation of VE-cadherin internalization and EC junctions. In light of the importance of PKM2 in cancer and other diseases such as diabetes, our findings provide information relevant to the development of PKM2-based therapeutic strategies.

## Experimental Procedures

### Antibodies and reagents

The following antibodies were used: PKM2 (#4053) and PKM1 (#7067) from Cell Signaling; β-actin (A441) and GAPDH (G9545) from Sigma; Rho-GDI (sc-360) and VE-cadherin (sc-6458) from Santa Cruz Biotechnologies; VE-cadherin (555289), plakoglobin (610254), GM130 (610605), and EEA1 (610685) from BD Pharmingen; Ki67 (IR626) from DAKO; and Ki67 (ab16667) from Abcam. F. Sánchez-Madrid (Centro Nacional de Investigaciones Cardiovasculares, CNIC, Madrid) kindly provided the anti-VE-cadherin TEA1/31 antibody^73^. IsolectinB4 (B-1205) was from Vector. Phalloidin, streptavidin, and Alexa Fluor secondary antibodies and Hoechst were from Life Technologies, and HRP secondary antibodies were from Jackson Immunoresearch Laboratories.

2-DG, ATP, FCCP, EDTA, glucose, luciferin, luciferase, mitomycin C, saponin, and shikonin were from Sigma Aldrich; cytodex3 from Amersham; fibrinogen, thrombin, and methylcellulose from Merck-Millipore; fluoromount-G from Southern Biotech; Ibidi chambers from Ibidi; phosphatase and proteinase inhibitors from Roche; Optiprep from AxisShield; and S1P from Cayman.

### Cell cultures

HUVECs (Lonza) were cultured in plastic dishes (Falcon and Ibidi) coated with 0.5% gelatin and were used for experiments at P3-P4. HUVECs were grown and maintained in 199 cultured medium (Lonza) supplemented with 20% FBS (Gibco), 100 U/ml penicillin, 100 µg/ml Streptomycin, 2 mM L-Glutamine, and 10 mM Hepes (all from Lonza), and ECGS-heparin factors (Promocell). For angiogenesis experiments, EGM-2 medium (Promocell) was used. HUVECs were serum-starved for at least 3 hours before experiments, and maintained without FBS throughout treatments.

Fibroblasts were provided by M. A. del Pozo (Centro Nacional de Investigaciones Cardiovasculares, CNIC, Madrid) and were cultured in IMDM medium supplemented with 10% FBS,100 U/ml penicillin, 100 µg/ml streptomycin, 1mM L-glutamine, 10 mM Hepes and 1 mM Na-pyruvate.

Mouse lung endothelial cells (MLECs) isolated from C57BL/6 mice and VE-cadherin-null mouse endothelial cells provided by E. Dejana (FIRC Institute of Molecular Oncology, Italy) were cultured as described previously^6^.

### Mouse retinal vasculature model

Retinal angiogenesis was assessed in C57BL/6 mice. Mice were housed and animal experiments performed under specific pathogen-free conditions at the Animal facility of Centro Nacional de Investigaciones Cardiovasculares Carlos III (CNIC), and in strict accordance with the institutional guidelines. Animal procedures were approved by the Committee on the Ethics of Animal Experiments of the CNIC (Permit Number: CNIC-01/13) and by the corresponding legal authority of the local government ‘Comunidad Autónoma’ of Madrid (Permit Number: PROEX 34/13). Animal studies were conformed to directive 2010/63EU and recommendation 2007/526/EC regarding the protection of animals used for experimental and other scientific purposes, enforced in Spanish law under RD1201/2005. Mice were fed ad libitum with standard chow (2108 Teklad global, Harlan Interfauna Iberica S.L.).

Animals were intravitreally injected with 1 µg of siRNA in Lipofectamine RNAimax at P3 and sacrificed at P6. For pharmacological inhibition of PKM2 activity, 100 µM of shikonin (or DMSO control) were injected at P5, and the mice were sacrificed at P6. Eyes were fixed overnight in 2% paraformaldehyde (PFA) and then enucleated, and retina was isolated as reported^74^.

### siRNA interference and plasmid and lentivirus transfection

Silencer™ Select siRNA oligonucleotides against isoform M2 of pyruvate kinase (Thermo Fisher) were transfected using Lipofectamine RNAimax (Thermo Fischer) in Optimem medium (Lonza) at a final siRNA concentration of 10 nM. Oligonucleotide sequences were 5′CCAUAAUCGUCCUCACCAA dTdT 3′ for human PKM2 and 5′ GUGCGAGCCUCCAGUCACU dTdT 3′ Ambion™ *In Vivo* for mouse PKM2. Control siRNA was from Ambion™ *In Vivo*.

Lentivirus containing Go-ATeam1^40^ was generated at VRC in Leuven Belgium or at the CNIC Viral Vectors Unit using the particles provided by Esteban Veiga-Chacón (Centro Nacional de Biotecnologia, CNB-CSIC, Madrid). Lentiviruses were added to cells at a M.O.I. of 10.

Lentivirus containing VE-Cadherin-EGFP and LifeAct-EGFP (kindly provided by Prof. Hans Schnittler from the Universität of Münster, Germany) was generated at the CNIC Viral Vectors Unit. Lentiviruses were added to cells at a M.O.I. of 10.

### Scratch assays

Confluent HUVECs were serum-starved for 3 hours and a wound was made with a plastic pipette tip. After washing the cells with PBS, migration was stimulated by adding medium containing 5% FBS. In the experiments in which proliferation was inhibited, cells were treated before and throughout the experiment with 2 μg ml^−1^ mitomycin C, 1 µM Shikonin or corresponding vehicle control. Migration was quantified as the proportion of the initial wound area remaining uncovered at the end of the experiment.

For collective migration, time-series pictures were taken every 20 minutes during 16 hours; only those experiments in which the endothelial monolayer remained confluent >80% after siRNA silencing or shikonin treatment were included in the analysis.

### Cell count assay

Cells were seeded at equal density in P96 wells. On each day of the experiment, cells in individual wells were detached and counted.

### Microbead spheroid assay

Cytodex3 microbeads were purchased from Amersham, and the protocol was performed as previously described^75^.

### Transendothelial electrical resistance (TEER)

TEER assays with an electric cell-substrate impedance sensing system (ECIS 1600R; Applied Biophysics^76^;) were performed as described^77,78^. The S1P-induced TEER increase was expressed as the percentage difference in resistance between unstimulated ECs and ECs stimulated with S1P for 3 hours.

### Immunofluorescence

Cells were fixed for 15 min in 4% PFA, blocked and permeabilized in 0.01% Triton X-100 2% BSA in PBS. Primary antibodies were incubated overnight in a cold room and secondary antibodies for 1 hour at 37 °C. Cell orientation was quantitated according to the relative position of Golgi versus nucleus toward or against the wound (±180°).

Fibrin gels were fixed in 1% PFA PBS for 30 minutes and washed. Gels were then permeabilized and blocked for 2 hours in blocking and permeabilization (BP) buffer (2% BSA, 0.3% Triton X-100, 0.3 M glycine, 0.2% azide, and 1% goat serum in PBS). Gels were incubated with primary antibodies overnight in the same buffer. The next day, gels were post-fixed in 4% PFA for 15 minutes. After a wash and 30 minutes in BP buffer, gels were incubated with secondary antibodies.

Retinas were blocked and fixed overnight in 2% BSA, 1% goat serum, 0.2% deoxycholate, 0.5% Triton X-100, and 0.2% azide in PBS. The next day, primary antibodies were added and incubations were maintained overnight. Retinas were then incubated overnight with Isolectin B4 in PBLEC buffer (1 mM MgCl_2_, 1 mM CaCl_2_, and 0.1 mM MnCl in PBS pH 6.8). Finally, secondary antibodies were added and incubations were maintained overnight. All preparations were mounted in Fluoromount-G.

### Proximity ligation assay (PLA)

HUVECs were grown to confluence, fixed and permeabilized. PLA was performed next day following the manufacturer’s instructions (Duolink PLA, Sigma Aldrich). Briefly, samples were blocked with the blocking solution for 30 min at 37 °C and incubated overnight with the primary antibodies, rabbit anti-PKM2 (#4053, Cell Signalling) and goat anti-VE-cadherin (sc-6458, Santa Cruz biotechnology), at 4 °C. The following day, the samples were washed and incubated for 1 h at 37 °C with PLA probes for rabbit and goat antibodies. After washing, samples were incubated with the ligase for 30 min at 37 °C, washed again and incubated with the polymerase for 100 min at 37 °C. Finally, the samples were washed, dried completely and mounted in Duolink *In Situ* Mounting Medium with Hoechst. Rabbit and goat IgGs were used as negative control.

### Cell live imaging

Live imaging was performed with a LSM700 Zeiss confocal microscope (Carl Zeiss) at a constant temperature of 37 °C and 5% CO_2_. PKM2-GFP was detected with 488 nm excitation and 510 nm emission. Go-ATeam1 was detected with 488 nm excitation and emission at 510 nm and 560 nm. VE-cadherin-GFP and LifeAct-GFP were detected with 488 nm excitation and 510 nm emission, time-series images were taken every minute for 15 minutes and after addition of 50 ng/ml of VEGFA for a further 15 min. Image analysis was performed using Fiji software.

### Membrane subfractionation

The membrane subfractionation protocol was performed as previously described^79^. Briefly, HUVEC membrane fractions were obtained and separated by ultracentrifugation on a discontinuous Optiprep density gradient (AxisShield). Protein fractions were precipitated, resuspended in Laemmli buffer, resolved by SDS-PAGE, and analyzed by western blot.

### Western blotting

Cells were lysed in RIPA buffer as described^6^. Retinas were isolated as reported^80^, and then RIPA buffer was added to the exposed vessels. Centrifugation was performed after 1 hour of incubation, and supernatant was recovered. Protein lysates were separated by 8% SDS-PAGE under reducing conditions. After transfer to a nitrocellulose membrane (Bio-Rad), blots were blocked with 5% BSA-PBS and incubated for 1 hour at room temperature (RT) with primary antibody diluted in 2% BSA-PBS. Membranes were then washed in 0.2% Tween 20-PBS and incubated for 1 hour at RT with horseradish peroxidase-conjugated secondary antibody. Protein bands were visualized by enhanced chemiluminescence (ECL; GE Healthcare). Band intensities were analyzed with Fiji software.

### Glucose and oxygen consumption

The extracellular acidification and oxygen consumption ratios (ECAR and OCR) were obtained in a Seahorse XF96 (Agilent), using glucose, oligomycin, FCCP and 2-DG, as described^81^.

### ATP levels

ATP production in saponin-enriched fractions was measured by a kinetic luciferin-luciferase luminescence assay, and normalized to the protein concentration in each sample^6^. Briefly, fractions were diluted in buffer A (150 mM KCl, 25 mM Tris-HCl pH 7.4, 2 mM EDTA, 0.1% BSA, 10 mM KPO4, 0.1 mM MgCl2) with 0.15 mM diadenosine pentaphosphate and then buffer B (0.5 M Tris–acetate pH 7.75, 0.8 mM luciferin, 20 μg/ml luciferase) and 0.1 mM ADP were added before recording light emission in the luminometer.

### Pyruvate kinase assay

PK activity was measured in saponin-permeabilized HUVEC fractions using a commercial kit from Biovision.

### VE-cadherin internalization

Internalization assays were performed as previously described^82^. Briefly, to measure internalization of endogenous VE-cadherin, confluent HUVEC were incubated with the VE-cadherin antibody (TEA1/31) at 4 °C for 1 h in 1% BSA medium. Then, we rinsed the cells in cold culture medium to remove unbound antibody and treated them with 50 ng/ml of VEGFA for 30 min at 37 °C to allow the internalization. Cells were acid-washed (2 mM PBS-glycine, pH 2.0) or washed with cold PBS 3 times for 10 min each (used as control) and fixed and processed for immunofluorescence.

### Confocal image quantification

Images were obtained with a Zeiss LSM700 confocal microscope (Carl Zeiss Microscopy) and maximum intensity projections are shown in all figures unless otherwise indicated. For analysis of mean fluorescence intensity, regions of interest (ROIs) were selected around junctions with Fiji software. For junction morphology analysis, we used reported criteria to distinguish discontinuous and continuous junctions^83^. For spheroid assays, mean length of sprouts and sprout number were analyzed in bright-field images with Fiji. Filopodia number was determined using phalloidin-stained F-actin. Data were normalized to cell length. For Go-aTeam1 analysis, the region of interest (ROI) for EC junctions was drawn ≤5 µm width around the cell-cell contacts defined by a 16 colors-pseudocolor plugin of Fiji software (validated by VE-cadherin staining). The cytosol ROI was defined as the rest of the cell.

Retinal parameters were quantified as described^74,84^. Radial growth was calculated as the average length in each lobule of one eye, from the optic nerve to the vascular front. Vascular density was calculated as the percentage of total area occupied by isolectin B4. Ki67 positive ECs were calculated as the percentage of Ki67 + ERG + cells of total ERG + cells, and normalized by Isolectin B4 area. Junction morphology was analyzed using the ‘patching algorithm’ implemented in MATLAB software^35^.

For VE-cadherin internalization experiments, images were taken using Nikon A1R confocal microscopy with a 40x objective and image analysis were performed using Fiji software.

For time-lapse scratch assay, images were taken with a Nikon time-lapse microscope with a 10x objective. Analysis of the tracking was done using Fiji with manual tracking plugin and Chemotaxis tool from ibidi.

### Statistical analysis

Absolute values were represented for most of the quantitated parameters. In cases of high variability in such values among experiments, fold induction versus control was calculated for each experiment as indicated in the corresponding legend. All values were analysed with Prism GraphPad. First all values were subjected to Grubbs’s outlier test (p < 0.05), and then D’Agostino-Pearson test for analysis of normal distribution was performed. The proper tests to compare samples were used as indicated in each figure legend. In general, for comparison of two samples, unpaired Student t-test was used in normal (Gaussian) data with equal variance, Welch’s test was used in normal (Gaussian) data with no equal variance and Mann-Whitney test for non-normal data; for comparison of three or more conditions, One-way ANOVA with Sidak-post test was used. Differences were considered significant at p < 0.05.

## Supplementary information

Supplementary Information

Movie S1

Movie S2

Movie S3

Movie S4

Movie S5

Movie S6

Movie S7

Movie S8

Movie S9

Movie S10

Movie S11

Movie S12

Movie S13

Movie S14

## Data Availability

All relevant data are available from the authors upon request.

## References

[1] Carmeliet P, Jain RK (2011). Molecular mechanisms and clinical applications of angiogenesis. Nature.

[2] Tung JJ, Tattersall IW, Kitajewski J (2012). Tips, stalks, tubes: notch-mediated cell fate determination and mechanisms of tubulogenesis during angiogenesis. Cold Spring Harb. Perspect. Med..

[3] Carmeliet P, De Smet F, Loges S, Mazzone M (2009). Branching morphogenesis and antiangiogenesis candidates: tip cells lead the way. Nat. Rev. Clin. Oncol..

[4] Serra H (2015). PTEN mediates Notch-dependent stalk cell arrest in angiogenesis. Nat Commun.

[5] Vitorino P (2015). MAP4K4 regulates integrin-FERM binding to control endothelial cell motility. Nature.

[6] Moreno V (2014). An EMMPRIN-gamma-catenin-Nm23 complex drives ATP production and actomyosin contractility at endothelial junctions. J. Cell Sci..

[7] Van Daele P, Van Coevorden A, Roger PP, Boeynaems JM (1992). Effects of adenine nucleotides on the proliferation of aortic endothelial cells. Circ. Res..

[8] Polet F, Feron O (2013). Endothelial cell metabolism and tumour angiogenesis: glucose and glutamine as essential fuels and lactate as the driving force. J. Intern. Med..

[9] DeBerardinis RJ (2010). & Cheng, T. Q’s next: the diverse functions of glutamine in metabolism, cell biology and cancer. Oncogene.

[10] Eelen G, de Zeeuw P, Simons M, Carmeliet P (2015). Endothelial cell metabolism in normal and diseased vasculature. Circ. Res..

[11] De Bock K (2013). Role of PFKFB3-driven glycolysis in vessel sprouting. Cell.

[12] Verdegem D, Moens S, Stapor P, Carmeliet P (2014). Endothelial cell metabolism: parallels and divergences with cancer cell metabolism. Cancer Metab.

[13] Yu P (2017). FGF-dependent metabolic control of vascular development. Nature.

[14] Eelen G (2018). Endothelial Cell Metabolism. Physiol. Rev..

[15] Rohlenova K, Veys K, Miranda-Santos I, De Bock K, Carmeliet P (2018). Endothelial Cell Metabolism in Health and Disease. Trends Cell Biol..

[16] Hu H (2016). Phosphoinositide 3-Kinase Regulates Glycolysis through Mobilization of Aldolase from the Actin Cytoskeleton. Cell.

[17] Jones DP (1986). Intracellular diffusion gradients of O2 and ATP. Am. J. Physiol..

[18] Green DE (1965). Association of integrated metabolic pathways with membranes. I. Glycolytic enzymes of the red blood corpuscle and yeast. Arch. Biochem. Biophys..

[19] Menard L, Maughan D, Vigoreaux J (2014). The structural and functional coordination of glycolytic enzymes in muscle: evidence of a metabolon?. Biology (Basel).

[20] Zala D (2013). Vesicular glycolysis provides on-board energy for fast axonal transport. Cell.

[21] Seidler NW (2013). Compartmentation of GAPDH. Adv. Exp. Med. Biol..

[22] Noguchi T, Inoue H, Tanaka T (1986). The M1- and M2-type isozymes of rat pyruvate kinase are produced from the same gene by alternative RNA splicing. J. Biol. Chem..

[23] Anastasiou D (2011). Inhibition of pyruvate kinase M2 by reactive oxygen species contributes to cellular antioxidant responses. Science.

[24] Mazurek S (2011). Pyruvate kinase type M2: a key regulator of the metabolic budget system in tumor cells. Int. J. Biochem. Cell Biol..

[25] Yamada K (1990). Tissue-specific expression of rat pyruvate kinase L/chloramphenicol acetyltransferase fusion gene in transgenic mice and its regulation by diet and insulin. Biochem. Biophys. Res. Commun..

[26] Christofk HR (2008). The M2 splice isoform of pyruvate kinase is important for cancer metabolism and tumour growth. Nature.

[27] Israelsen WJ (2013). PKM2 isoform-specific deletion reveals a differential requirement for pyruvate kinase in tumor cells. Cell.

[28] Lunt SY (2015). Pyruvate kinase isoform expression alters nucleotide synthesis to impact cell proliferation. Mol. Cell.

[29] Hosios AM, Fiske BP, Gui DY, Vander Heiden MG (2015). Lack of Evidence for PKM2 Protein Kinase Activity. Mol. Cell.

[30] Lu Z, Hunter T (2018). Metabolic Kinases Moonlighting as Protein Kinases. Trends Biochem. Sci..

[31] Li L, Zhang Y, Qiao J, Yang JJ, Liu ZR (2014). Pyruvate kinase M2 in blood circulation facilitates tumor growth by promoting angiogenesis. J. Biol. Chem..

[32] Boeckel JN (2016). JMJD8 Regulates Angiogenic Sprouting and Cellular Metabolism by Interacting With Pyruvate Kinase M2 in Endothelial Cells. Arterioscler. Thromb. Vasc. Biol..

[33] Lee OH (1999). Sphingosine 1-phosphate induces angiogenesis: its angiogenic action and signaling mechanism in human umbilical vein endothelial cells. Biochem. Biophys. Res. Commun..

[34] Puchulu-Campanella E (2013). Identification of the components of a glycolytic enzyme metabolon on the human red blood cell membrane. J. Biol. Chem..

[35] Bentley K (2014). The role of differential VE-cadherin dynamics in cell rearrangement during angiogenesis. Nat. Cell Biol..

[36] Hayer A (2016). Engulfed cadherin fingers are polarized junctional structures between collectively migrating endothelial cells. Nat. Cell Biol..

[37] Martin, M., Veloso, A., Wu, J., Katrukha, E. A. & Akhmanova, A. Control of endothelial cell polarity and sprouting angiogenesis by non-centrosomal microtubules. *Elife* **7**, 10.7554/eLife.33864 (2018). 10.7554/eLife.33864 PMC5898915 29547120

[38] Millarte V, Farhan H (2012). The Golgi in cell migration: regulation by signal transduction and its implications for cancer cell metastasis. ScientificWorldJournal.

[39] Chen J (2011). Shikonin and its analogs inhibit cancer cell glycolysis by targeting tumor pyruvate kinase-M2. Oncogene.

[40] Nakano M, Imamura H, Nagai T, Noji H (2011). Ca(2)(+) regulation of mitochondrial ATP synthesis visualized at the single cell level. ACS Chem. Biol..

[41] Cavey M, Lecuit T (2009). Molecular bases of cell-cell junctions stability and dynamics. Cold Spring Harb. Perspect. Biol..

[42] Cao Jiahui, Schnittler Hans (2019). Putting VE-cadherin into JAIL for junction remodeling. Journal of Cell Science.

[43] West JJ, Harris TJ (2016). Cadherin Trafficking for Tissue Morphogenesis: Control and Consequences. Traffic.

[44] Xiao K (2005). p120-Catenin regulates clathrin-dependent endocytosis of VE-cadherin. Mol. Biol. Cell.

[45] Dayton TL (2016). Germline loss of PKM2 promotes metabolic distress and hepatocellular carcinoma. Genes Dev..

[46] Kim B (2018). Endothelial pyruvate kinase M2 maintains vascular integrity. J. Clin. Invest..

[47] Stone OA (2018). Loss of pyruvate kinase M2 limits growth and triggers innate immune signaling in endothelial cells. Nat Commun.

[48] Chen YL (2015). Mechanisms of pyruvate kinase M2 isoform inhibits cell motility in hepatocellular carcinoma cells. World J. Gastroenterol..

[49] Wang C (2017). PKM2 promotes cell migration and inhibits autophagy by mediating PI3K/AKT activation and contributes to the malignant development of gastric cancer. Sci. Rep..

[50] Yang P (2015). Secreted pyruvate kinase M2 facilitates cell migration via PI3K/Akt and Wnt/beta-catenin pathway in colon cancer cells. Biochem. Biophys. Res. Commun..

[51] Ding H (2014). Phosphoglycerate kinase 1 promotes radioresistance in U251 human glioma cells. Oncol. Rep..

[52] Delva E, Kowalczyk AP (2009). Regulation of cadherin trafficking. Traffic.

[53] Huang RL (2011). ANGPTL4 modulates vascular junction integrity by integrin signaling and disruption of intercellular VE-cadherin and claudin-5 clusters. Blood.

[54] Onodera Y, Nam JM, Bissell MJ (2014). Increased sugar uptake promotes oncogenesis via EPAC/RAP1 and O-GlcNAc pathways. J. Clin. Invest..

[55] Fantin A (2015). NRP1 Regulates CDC42 Activation to Promote Filopodia Formation in Endothelial Tip Cells. Cell Rep.

[56] Phng LK, Stanchi F, Gerhardt H (2013). Filopodia are dispensable for endothelial tip cell guidance. Development.

[57] De Bock K, Georgiadou M, Carmeliet P (2013). Role of endothelial cell metabolism in vessel sprouting. Cell Metab.

[58] Dorland YL (2016). The F-BAR protein pacsin2 inhibits asymmetric VE-cadherin internalization from tensile adherens junctions. Nat Commun.

[59] Huveneers S (2012). Vinculin associates with endothelial VE-cadherin junctions to control force-dependent remodeling. J. Cell Biol..

[60] Neto, F. *et al*. YAP and TAZ regulate adherens junction dynamics and endothelial cell distribution during vascular development. *Elife* **7**, 10.7554/eLife.31037 (2018). 10.7554/eLife.31037 PMC5814147 29400648

[61] Cantelmo AR (2016). Inhibition of the Glycolytic Activator PFKFB3 in Endothelium Induces Tumor Vessel Normalization, Impairs Metastasis, and Improves Chemotherapy. Cancer Cell.

[62] Israelsen WJ, Vander Heiden MG (2015). Pyruvate kinase: Function, regulation and role in cancer. Semin. Cell Dev. Biol..

[63] Kaltenbach LS (2007). Huntingtin interacting proteins are genetic modifiers of neurodegeneration. PLoS Genet.

[64] Hinckelmann MV (2016). Self-propelling vesicles define glycolysis as the minimal energy machinery for neuronal transport. Nat Commun.

[65] Yang W (2011). Nuclear PKM2 regulates beta-catenin transactivation upon EGFR activation. Nature.

[66] Fraccaroli A (2015). Endothelial alpha-parvin controls integrity of developing vasculature and is required for maintenance of cell-cell junctions. Circ. Res..

[67] Wimmer R, Cseh B, Maier B, Scherrer K, Baccarini M (2012). Angiogenic sprouting requires the fine tuning of endothelial cell cohesion by the Raf-1/Rok-alpha complex. Dev. Cell.

[68] Li J (2016). The Poly-cistronic miR-23-27-24 Complexes Target Endothelial Cell Junctions: Differential Functional and Molecular Effects of miR-23a and miR-23b. Mol Ther Nucleic Acids.

[69] Cruys B (2016). Glycolytic regulation of cell rearrangement in angiogenesis. Nat Commun.

[70] Ubezio, B. *et al*. Synchronization of endothelial Dll4-Notch dynamics switch blood vessels from branching to expansion. *Elife* **5**, 10.7554/eLife.12167 (2016). 10.7554/eLife.12167 PMC4894757 27074663

[71] Conradi LC (2017). Tumor vessel disintegration by maximum tolerable PFKFB3 blockade. Angiogenesis.

[72] Maeda T., Inagaki M., Fujita Y., Kimoto T., Tanabe-Fujimura C., Zou K., Liu J., Liu S., Komano H. (2016). ATP increases the migration of microglia across the brain endothelial cell monolayer. Bioscience Reports.

[73] Galvez BG, Matias-Roman S, Albar JP, Sanchez-Madrid F, Arroyo AG (2001). Membrane type 1-matrix metalloproteinase is activated during migration of human endothelial cells and modulates endothelial motility and matrix remodeling. J. Biol. Chem..

[74] Pitulescu ME, Schmidt I, Benedito R, Adams RH (2010). Inducible gene targeting in the neonatal vasculature and analysis of retinal angiogenesis in mice. Nat. Protoc..

[75] Nakatsu MN (2003). Angiogenic sprouting and capillary lumen formation modeled by human umbilical vein endothelial cells (HUVEC) in fibrin gels: the role of fibroblasts and Angiopoietin-1. Microvasc. Res..

[76] Tiruppathi C, Malik AB, Del Vecchio PJ, Keese CR, Giaever I (1992). Electrical method for detection of endothelial cell shape change in real time: assessment of endothelial barrier function. Proc. Natl. Acad. Sci. USA.

[77] Aranda JF (2013). MYADM controls endothelial barrier function through ERM-dependent regulation of ICAM-1 expression. Mol. Biol. Cell.

[78] Fernandez-Martin L (2012). Crosstalk between reticular adherens junctions and platelet endothelial cell adhesion molecule-1 regulates endothelial barrier function. Arterioscler. Thromb. Vasc. Biol..

[79] Manickam V (2011). Regulation of vascular endothelial growth factor receptor 2 trafficking and angiogenesis by Golgi localized t-SNARE syntaxin 6. Blood.

[80] Choi JH (2018). mTORC1 accelerates retinal development via the immunoproteasome. Nat Commun.

[81] Rose S (2014). Oxidative stress induces mitochondrial dysfunction in a subset of autism lymphoblastoid cell lines in a well-matched case control cohort. PLoS One.

[82] Orsenigo F (2012). Phosphorylation of VE-cadherin is modulated by haemodynamic forces and contributes to the regulation of vascular permeability *in vivo*. Nat Commun.

[83] Millan J (2010). Adherens junctions connect stress fibres between adjacent endothelial cells. BMC Biol..

[84] Wilhelm K (2016). FOXO1 couples metabolic activity and growth state in the vascular endothelium. Nature.

