# Supplementary material for: PKM2 regulates endothelial cell junction dynamics and angiogenesis via ATP production
Source: Sci Rep. 2019 Oct 21;9:15022. doi: 10.1038/s41598-019-50866-x (PMC6803685; doi:10.1038/s41598-019-50866-x)
Supplement: Supplementary file 1 — Supplementary Information [file 41598_2019_50866_MOESM1_ESM.pdf]

Supplementary Information

PKM2 regulates endothelial cell junction dynamics and angiogenesis via ATP production

Jesús Gómez-Escudero, Cristina Clemente, Diego García-Weber, Rebeca Acín-Pérez, Jaime Millán, José A. Enríquez, Katie Bentley, Peter Carmeliet, and Alicia G. Arroyo

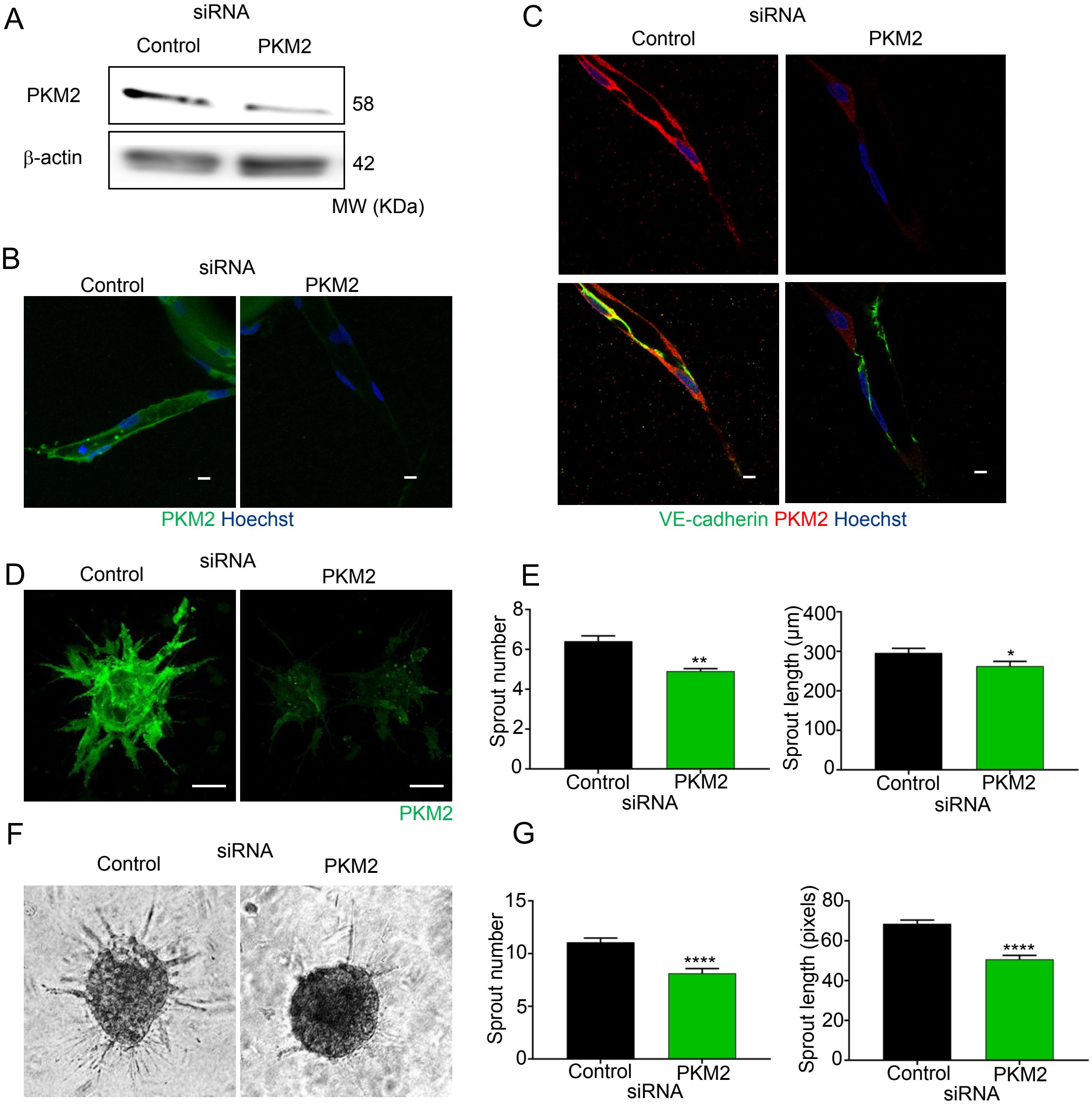

Supp Figure S1. Impact of PKM2 silencing in endothelial cells in 3D spheroid sproutings asays. (A) Western blot of PKM2 in HUVECs transfected with control or PKM2 siRNA at the final day of the spheroid-sprouting assay. β-actin is included as a loading control. MW, molecular weight. (B) Immunofluorescence of PKM2 (green) and Hoechst (blue, nuclei) in sprouts of 3D EC spheroids at the final day. Scale bar, 10 μm. (C) Immunofluorescence of PKM2 (red), VE-cadherin (green), and Hoechst (blue,nuclei) in 3D spheroid sprouts shows a clear lumen. Scale bar, 10 μm. (D) Immunofluorescence of PKM2 (green) in spheroids formed by control and PKM2-silenced HUVEC embedded and grown in fibrin gels for 24 hours. Scale bar, 50 μm. (E) Sprout number and length in 3D EC spheroids from D; means ± SEM, n=5 independent experiments, *$p<0.05$ and **$p<0.01$ by paired Student t-test. (F) Images of 3D spheroids formed by mouse lung endothelial cells (MLEC) silenced with control or PKM2 siRNA at the final day of the spheroid-sprouting assay. (G) Sprout number and length in 3D EC spheroids from F; means ± SEM, n=3 independent experiments, ****$p<0.0001$ by Mann-Whitney test.

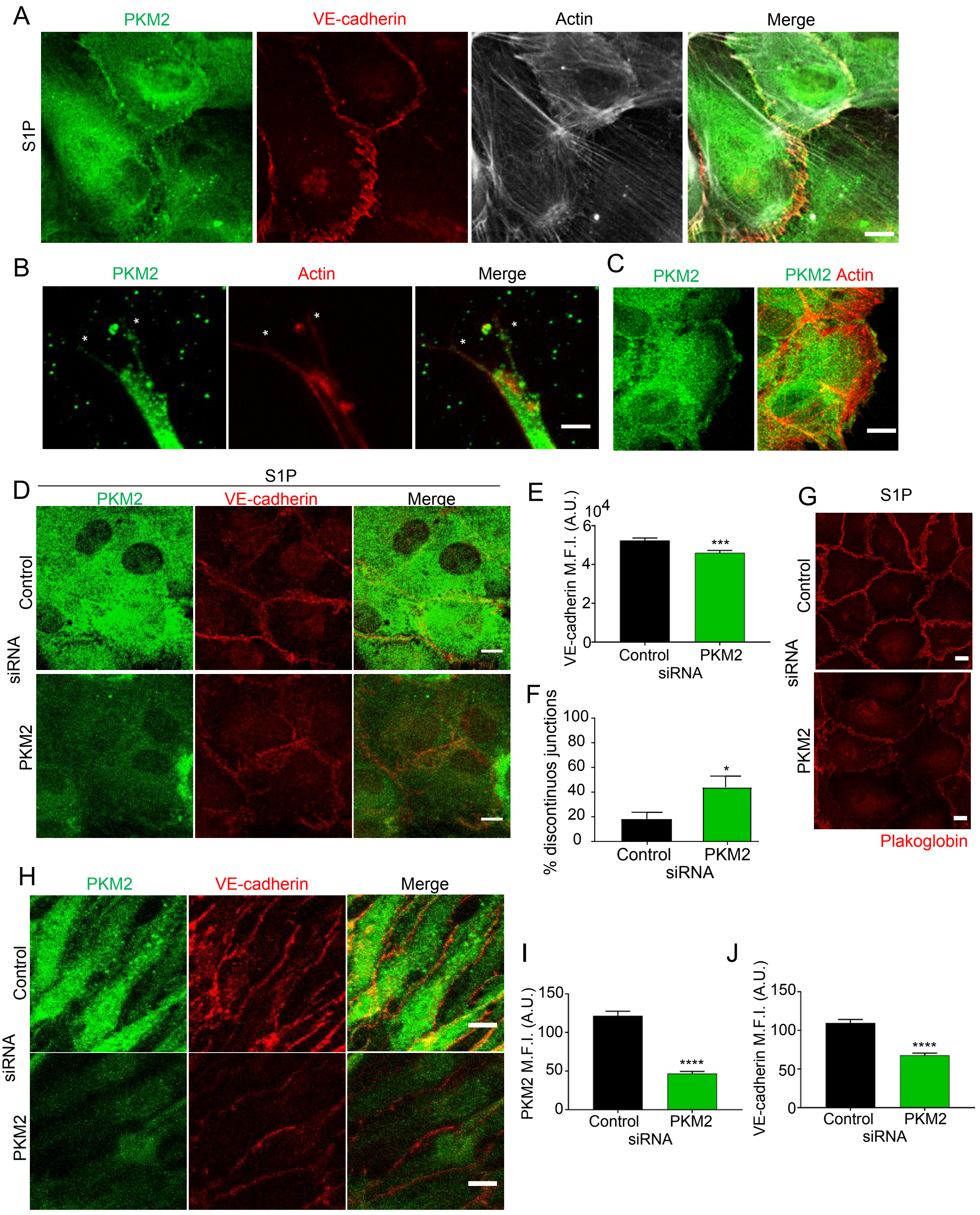

A
PKM2
VE-cadherin
Actin
Merge
S1P
B
PKM2
Actin
Merge
C
PKM2
PKM2 Actin
D
S1P
PKM2
VE-cadherin
Merge
Control
siRNA
PKM2
E
VE-cadherin M.F.I. (A.U.)
10^4
6
4
2
0
***
Control
PKM2
siRNA
F
% discontinuos junctions
100
80
60
40
20
0
*
Control
PKM2
siRNA
G
S1P
Control
siRNA
PKM2
Plakoglobin
H
PKM2
VE-cadherin
Merge
Control
siRNA
PKM2
I
PKM2 M.F.I. (A.U.)
150
100
50
0
****
Control
PKM2
siRNA
J
VE-cadherin M.F.I. (A.U.)
150
100
50
0
****
Control
PKM2
siRNA

Supp Figure S2. PKM2 is present at F-actin membrane protrusions and at remodeling EC junctions. (A) Immunofluorescence of PKM2 (green), VE-cadherin (red) and F-actin (grey) in confluent HUVECs treated with 1 µM S1P for 15 minutes; single color and merged images are shown. Scale bar, 10 µm. (B) Immunofluorescence of PKM2 (green), F-actin (red), and Hoechst (blue, nuclei) of sprout filopodia in 3D EC spheroids. Scale bar, 5 µm. Asterisks indicate individual filopodium. (C) Immunofluorescence of PKM2 (green) and F-actin (red) in subconfluent migrating HUVECs. Scale bar, 10 µm. (D) Immunofluorescence of VE-cadherin (red) and PMK2 (green) in confluent HUVECs transfected with control or PKM2 siRNA and treated with 1 µM S1P for 15 minutes. Scale bar, 10 µm. (E) VE-cadherin mean fluorescence intensity (M.F.I.) at junctions of control and PKM2 silenced HUVECs treated as in D; means ± SEM, n= 5 independent experiments, ***$p<0.001$ by Mann-Whitney test. (F) Percentage of VE-cadherin discontinuous junctions; means ± SEM, n=3 independent experiments, *$p<0.05$ by Mann-Whitney test. (G) Immunofluorescence of plakoglobin (red) in confluent HUVECs transfected with control or PKM2 siRNA and treated with 1 µM S1P for 15 minutes. Scale bar, 10 µm. (H) Immunofluorescence of PKM2 (green) and VE-cadherin (red) in confluent mouse lung endothelial cells (MLECs) transfected with control or PKM2 siRNA. Scale bar, 25 µm. (I and J) PKM2 (I) and VE-cadherin (J) mean fluorescence intensity (M.F.I.) at junctions of control and PKM2-silenced MLECs; means ± SEM, n=3 independent experiments, ****$p<0.001$ by Mann-Whitney test.

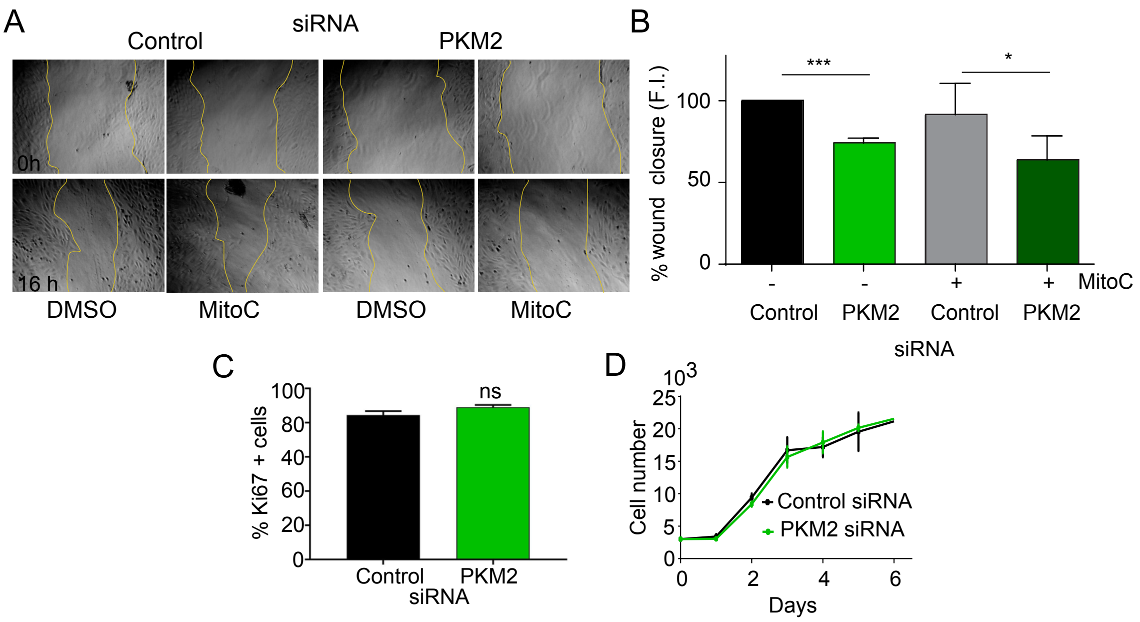

Supp Figure S3. PKM2 silencing does not impact proliferation in migrating endothelial cells. (A) Bright-field microscopy images of siRNA-silenced HUVECs at time 0 and 16 hours after scratch wounding. Cells were treated during the assay with vehicle (DMSO) or 2 μg ml^{-1} mitomycin C (MitoC). (B) Percentage of wound closure 16 hours after scratch wounding quantified as fold induction (F.I.) versus control; means ± SEM, n=4 independent experiments. *$p<0.05$ and ***$p<0.001$ by one-way ANOVA with Sidak post-test. (C) Percentage of Ki67-positive cells in confluent HUVECs transfected with control or PKM2 siRNA; means ± SEM, n=3 independent experiments, ns non-significant by unpaired Student t-test. (D) Time course of the number of control and PKM2-silenced HUVECs; means ± SEM, n=4 independent experiments.

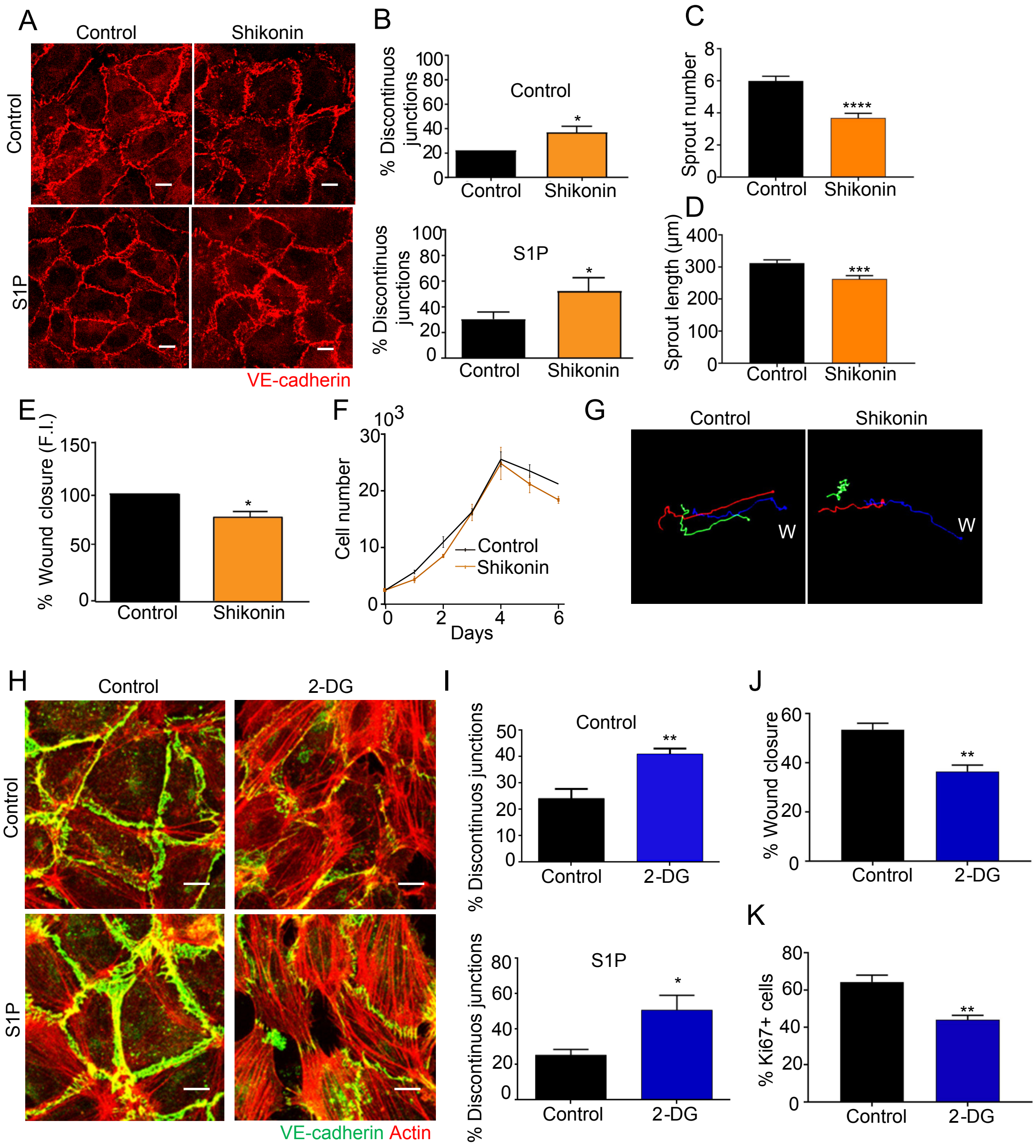

A
Control
Shikonin
Control
S1P
VE-cadherin
B
% Discontinuos junctions
Control
Control
Shikonin
S1P
C
Sprout number
****
Control
Shikonin
D
Sprout length (µm)
***
E
% Wound closure (F.I.)
F
Cell number
10^3
Days
Control
Shikonin
G
Control
Shikonin
W
H
Control
2-DG
S1P
VE-cadherin Actin
I
% Discontinuos junctions
J
% Wound closure
K
% Ki67+ cells

Supp Figure S4. Inhibition of PKM2 activity recapitulates the *in vitro* phenotype of PKM2-silenced ECs. (A) Immunofluorescence of VE-cadherin (red) in HUVECs treated with 1 μM shikonin or DMSO (control) and stimulated or not with 1 μM S1P for 15 minutes. Scale bar, 10 μm.(B) Percentage of discontinuous VE-cadherin junctions in the different conditions; means ± SEM, n=3 independent experiments, *p<0.05 by unpaired Student t-test.(C and D) Sprout number and length in 3D spheroids formed by DMSO- and shikonin-treated HUVEC; means ± SEM, n=3 independent experiments with at least 20 spheroids analyzed per experiment, ***p<0.001 and ****p<0.0001 by unpaired Student t-test. (E) Percentage of wound closure by HUVECs treated with 1μM shikonin or DMSO 16 hours after scratch wounding, quantified as fold induction (F.I.) versus control; means ± SEM, n=4 independent experiments. *p<0.05 by paired Student t-test. (F) Time course of HUVEC number in cultures treated with 1 μM shikonin or DMSO (control); means ± SEM, n=4 independent experiments.(G) Single-cell tracks of three adjacent HUVECs treated with 1 μM shikonin or DMSO migrating during the scratch assay. (H) Immunofluorescence of VE-cadherin (green) and F-actin (red) in HUVECs treated with 2 mM 2-DG or vehicle (control) and stimulated or not with 1 μM S1P for 15 minutes. Scale bar, 10 μm. (I) Percentage of discontinuous VE-cadherin-junctions in the different conditions; means ± SEM, n=3 independent experiments, *p<0.05 and **p<0.01 by unpaired Student t-test. (J) Percentage of wound closure by HUVECs treated or not with 2 mM 2-deoxyglucose (2-DG) 16 hours after scratch wounding; means ± SEM, n=3 independent experiments, **p<0.01 by unpaired Student-test. (K) Percentage of Ki67-positive cells in HUVECs treated or not with 2 mM 2-deoxyglucose 2-DG); means ± SEM, n=3 independent experiments, **p<0.01 by Mann Whitney test. See related movies S3 and S4.

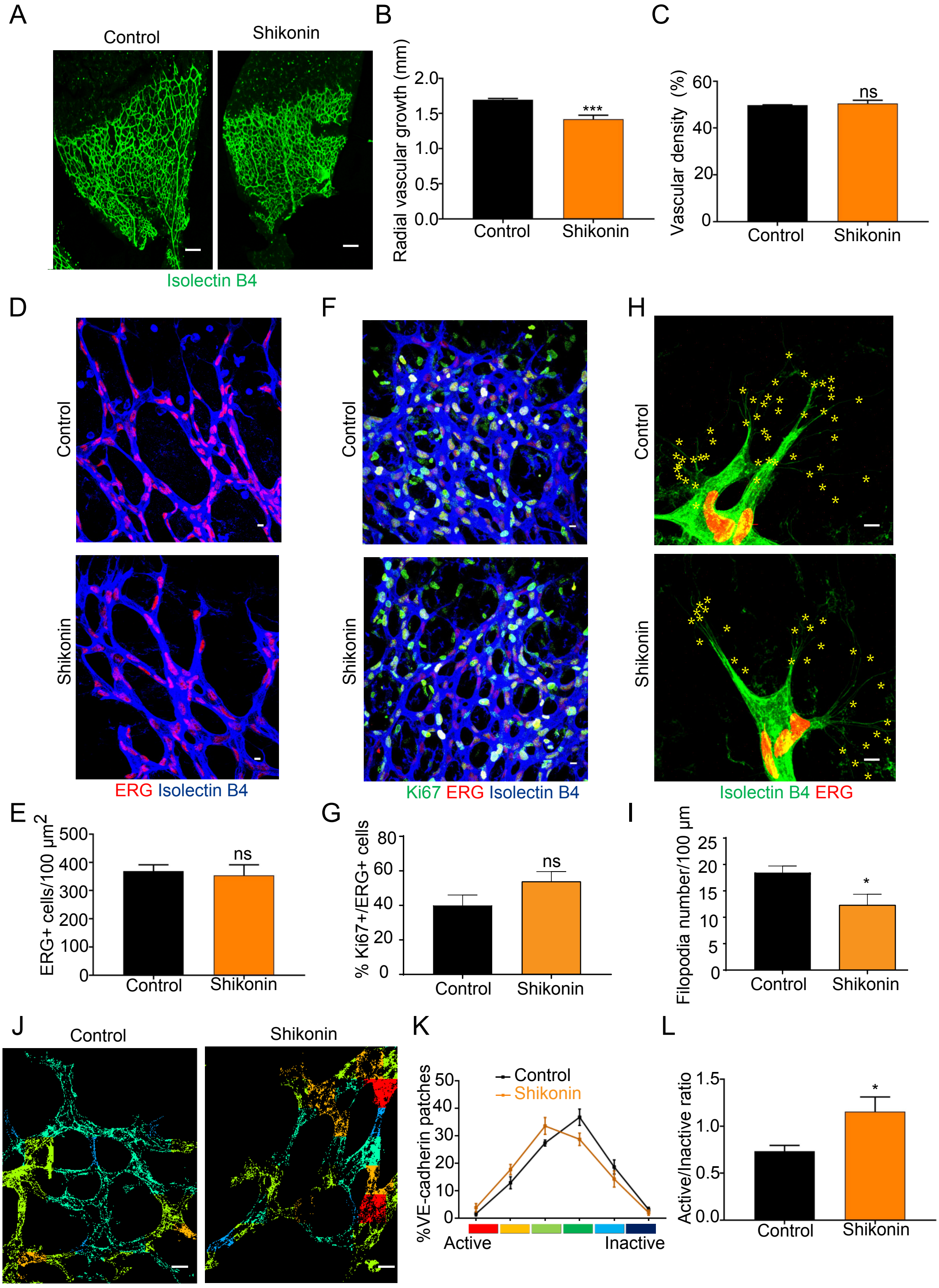
A
Control
Shikonin
Isolectin B4
B
Radial vascular growth (mm)
2.0
1.5
1.0
0.5
0.0
***
Control
Shikonin
C
Vascular density (%)
60
40
20
0
ns
Control
Shikonin
D
Control
Shikonin
ERG Isolectin B4
F
Control
Shikonin
Ki67 ERG Isolectin B4
H
Control
Shikonin
Isolectin B4 ERG
E
ERG+ cells/100 μm2
500
400
300
200
100
0
ns
Control
Shikonin
G
% Ki67+/ERG+ cells
80
60
40
20
0
ns
Control
Shikonin
I
Filopodia number/100 μm
25
20
15
10
5
0
*
Control
Shikonin
J
Control
Shikonin
K
%VE-cadherin patches
50
40
30
20
10
0
Control
Shikonin
Active
Inactive
L
Active/Inactive ratio
1.5
1.0
0.5
0.0
*
Control
Shikonin

Supp Figure S5. PKM2 activity is required for angiogenesis *in vivo*. (A) Immunofluorescence of isolectin B4 (green, vessels) in P6 mouse whole-mount retinas 24 hours after intravitreal injection of 100 µM shikonin or DMSO (control). Scale bar, 50 µm. (B) Radial vascular growth in P6 mouse retinas treated as in A; means ± SEM, n=6 mice per condition. ***$p<0.001$ by Mann-Whitney test. (C) Vascular density in P6 mouse retinas treated as in A; means ± SEM, n=4 mice per condition, ns non-significant by Mann-Whitney test. (D) Immunofluorescence of ERG (red, endothelial cell nuclei) and isolectin B4 (blue, vessels) in P6 mouse whole-mount retinas 24 hours after intravitreal injection of 100 µM shikonin or DMSO (control). Scale bar, 10 µm. (E) ERG-positive cells per vessel area in P6 mouse retinas treated as in D; means ± SEM, n=4 mice, ns non-significant by unpaired Student t-test. (F) Immunofluorescence of Ki67 (green, proliferation), ERG (red, endothelial cell nuclei), and isolectin B4 (blue, vessels) in P6 mouse whole-mount retinas 24 hours after intravitreal injection of 100 µM shikonin or DMSO (control). Scale bar, 10 µm. (G) Percentage of Ki67-positive cells per ERG-positive cells in P6 mouse retinas treated as in F; means ± SEM, n=4 mice, *$p<0.05$ by unpaired Student t-test. (H) Immunofluorescence ERG (red) and isolectin B4 (blue, vessels) in P6 mouse whole-mount retinas 24 hours after intravitreal injection of 100 µM shikonin or DMSO (control). Yellow arrows mark filopodia. Scale bar, 10 µm. (I) Number of filopodia per 100 µm of vascular front in P6 mouse retinas treated as in H; means ± SEM, n=4 mice. *$p<0.05$ by unpaired Student t-test. (J) Pseudo-colored images according to the patching algorithm classification of active/inactive junctions from VE-cadherin whole-mount stained P6 mouse retinas 24 hours after intravitreal injection of 100 µM shikonin or DMSO (control). Scale bar, 10 µm. (K and L) Percentage of VE-cadherin active/inactive junction patches (K) and their ratio (L) in P6 mouse retinas treated as in J; means ± SEM, n=4 mice, *$p<0.05$ by unpaired Student t-test.

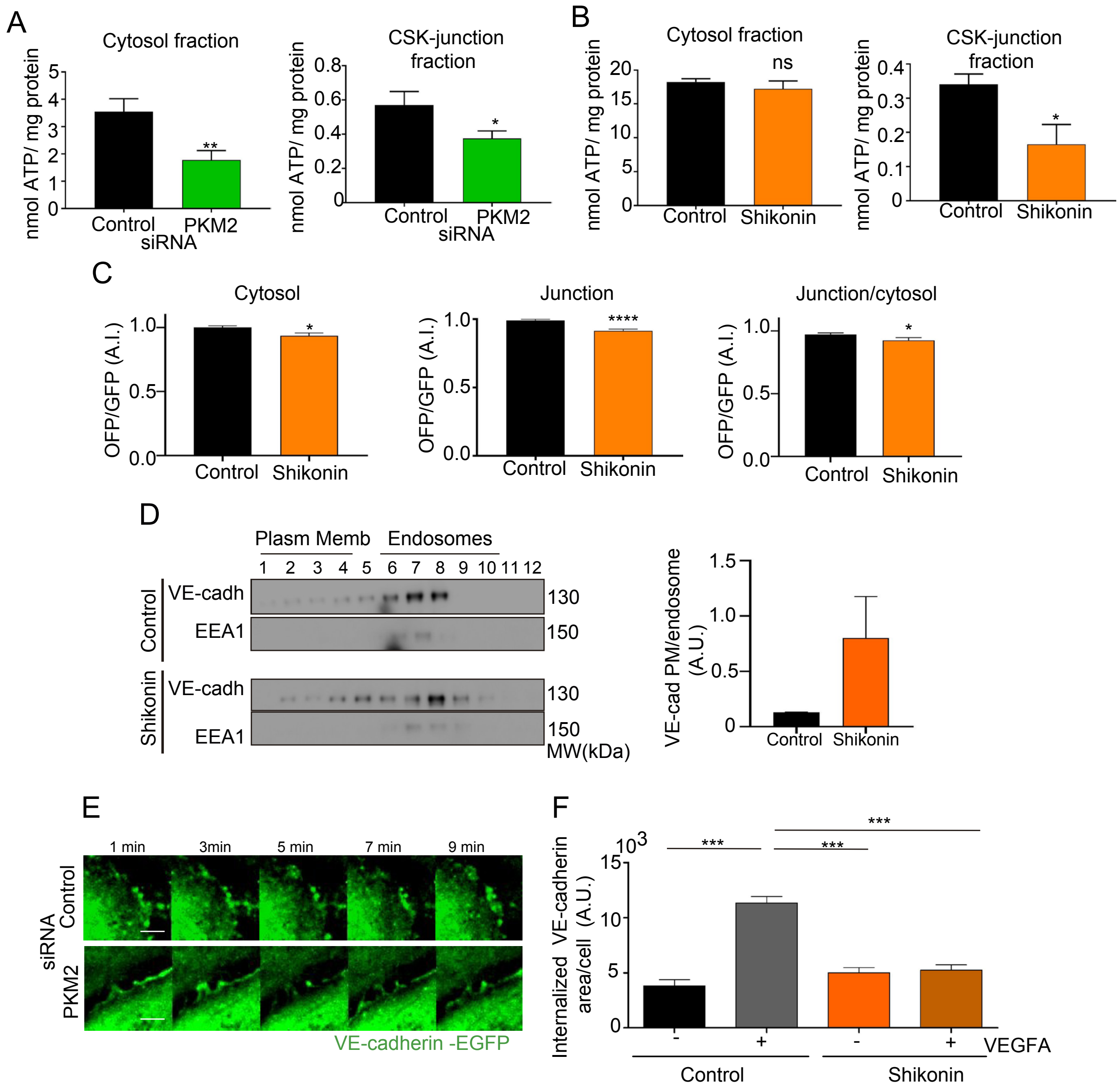

Supp Figure S6. PKM2 activity is required for ATP production at EC junctions and VE-cadherin internalization. (A) ATP amount normalized to protein content in the cytosol and CSK-junction-enriched fractions extracted sequentially with 0.01% and 0.1% saponin (as in Figure 6H) from mouse lung endothelial cells transfected with control or PKM2 siRNA; means ± SEM, n=11-12 independent samples, *p<0.05 and **p<0.01 by unpaired Student-test. (B) ATP amount normalized to protein content in the cytosol and CSK-junction-enriched fractions extracted as in A from HUVECs treated with 1 µM shikonin or DMSO (control); means ± SEM, n=6-7 independent samples, ns non-significant and *p<0.05 by unpaired Student t-test. (C) OFP:GFP intensity ratio at the cytosol, junctions and junction/cytosol of GO-ATeam1-transduced HUVECs treated with 1 µM shikonin or DMSO (control); means ± SEM, n=4 independent experiments, *p<0.05 and ****p<0.0001 by Mann-Whitney test. (D) Western blot of VE-cadherin in gradient density fractions of membranes from HUVECs treated with shikonin or vehicle. EEA1 is included as a marker of endosomes. Ratio of VE-cadherin expression in plasma membrane (PM) fractions versus endosomes; n=2 independent experiments. MW, molecular weight. (E) Still images from live microscopy of HUVECs transduced with lentivirus coding for VE-cadherin-EGFP; siControl and siPKM2-silenced cells are shown. Scale bar, 5 µ m. (F) Internalized VE-cadherin positive area per cell in arbitrary units (A.U.) from HUVECs treated with 1 µM shikonin or DMSO (control); means ± SEM, n=3 independent experiments **p<0.01 and ***p<0.001 by one-way ANOVA with Sidak post-test. See related Movies S9-S10.

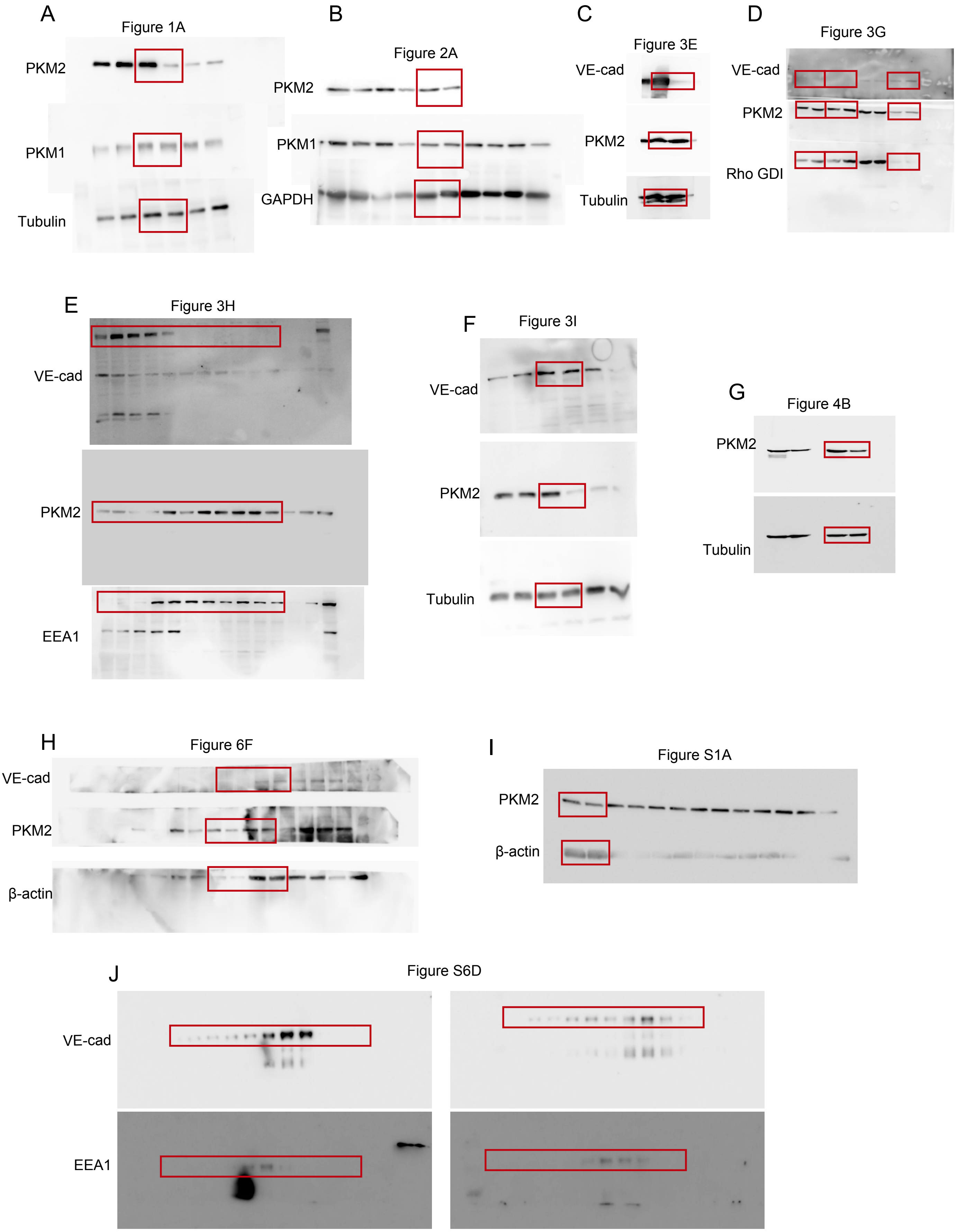

Supp Figure S7. Uncropped western blots used for Main and Supplementary Figures. Red boxes indicate the bands that appear in each Figure.

Supplementary movies

Movie S1. PKM2 expression is required for endothelial cell collective migration, Related to Figure 4.

Time-lapse movie of control siRNA-silenced HUVECs showing their collective migrating during 16 hours after the monolayer was scratched. Cells do not migrate as directionally toward the wound and EC collective migration looks impaired compared to controls. Phase-contrast images were taken every 20 min. Elapsed time is designated as hh:mm. Bar, 100 μm.

Movie S2. PKM2 expression is required for endothelial cell collective migration, Related to Figure 4.

Time-lapse movie of PKM2 siRNA-silenced HUVECs showing their collective migrating during 16 hours after the monolayer was scratched. Phase-contrast images were taken every 20 min. Elapsed time is designated as hh:mm. Bar, 100 μm.

Movie S3. PKM2 activity is required for endothelial cell collective migration, Related to Supplementary Figure S4.

Time-lapse movie of vehicle-treated HUVECs collectively migrating during 16 hours after the monolayer was scratched. Phase-contrast images were taken every 20 min. Elapsed time is designated as hh:mm. Bar, 100 μm.

Movie S4. PKM2 activity is required for endothelial cell collective migration, Related to Supplementary Figure S4.

Time-lapse movie of shikonin-treated HUVECs collectively migrating during 16 hours after the monolayer was scratched. Cells do not migrate as directionally toward the wound and EC collective migration looks impaired compared to controls. Phase-contrast images were taken every 20 min. Elapsed time is designated as hh:mm. Bar, 100 μm.

Movie S5. PKM2 inhibition increases intercellular gaps and reduced VE-cadherin plaques at the endothelial junctions, Related to Figure 7.

Time-lapse movie of vehicle-treated HUVECs transduced with VE-cadherin-GFP lentivirus showing VE-cadherin dynamics at the endothelial cell junctions. Fluorescence images were taken every 1 minute. Elapsed time is designated as hh:mm. Bar, 10 μm.

Movie S6. PKM2 inhibition increases intercellular gaps and reduced VE-cadherin plaques at the endothelial junctions, Related to Figure 7.

Time-lapse movie of vehicle-treated HUVECs transduced with VE-cadherin-GFP lentivirus and treated with 50 ng/ml of VEGF showing opening of intercellular gaps, and increased number of VE-cadherin plaques at the endothelial cell junctions compared to untreated cells. Fluorescence images were taken every 1 minute. Elapsed time is designated as hh:mm. Bar, 10 μm.

Movie S7. PKM2 inhibition increases intercellular gaps and reduced VE-cadherin plaques at the endothelial junctions, Related to Figure 7.
Time-lapse movie of shikonin-treated HUVECs transduced with VE-cadherin-GFP lentivirus showing increased number of intercellular gaps and decreased VE-cadherin dynamics at the endothelial cell junctions compared to controls. Fluorescence images were taken every 1 minute. Elapsed time is designated as hh:mm. Bar, 10 μm.

Movie S8. PKM2 inhibition increases intercellular gaps and reduced VE-cadherin plaques at the endothelial junctions, Related to Figure 7.
Time-lapse movie of shikonin-treated HUVECs transduced with VE-cadherin-GFP lentivirus and treated with 50 ng/ml of VEGF showing opening of abundant intercellular gaps, and reduced number of VE-cadherin plaques at the endothelial cell junctions compared to controls. Fluorescence images were taken every 1 minute. Elapsed time is designated as hh:mm. Bar, 10 μm.

Movie S9. PKM2 expression is required for VE-cadherin junction stability, Related to Supplementary Figure S6E.
Time-lapse movie of control siRNA-silenced HUVECs, transduced with VE-cadherin-GFP lentivirus showing stable VE-cadherin endothelial cell junctions. Fluorescence images were taken every 1 minute. Elapsed time is designated as hh:mm. Bar, 10 μm.

Movie S10. PKM2 expression is required for VE-cadherin junction stability, Related to Supplementary Figure S6E.
Time-lapse movie of PKM2 siRNA-silenced HUVECs, transduced with VE-cadherin-GFP lentivirus showing increased number of intercellular gaps and decreased VE-cadherin dynamics at the endothelial cell junctions compared to controls. Fluorescence images were taken every 1 minute. Elapsed time is designated as hh:mm. Bar, 10 μm.

Movie S11. PKM2 inhibition decreases the number of ‘junction-associated intermittent lamellipodia’ (JAIL) at the endothelial junctions, Related to Figure 7.

Time-lapse movie of vehicle-treated HUVECs transduced with LifeAct-GFP lentivirus showing F-actin dynamics at the endothelial cell junctions. Fluorescence images were taken every 1 minute. Elapsed time is designated as hh:mm. Bar, 10 μm.

Movie S12. PKM2 inhibition decreases the number of 'junction-associated intermittent lamellipodia' (JAIL) at the endothelial junctions, Related to Figure 7.

Time-lapse movie of vehicle-treated HUVECs transduced with LifeAct-GFP lentivirus and treated with 50 ng/ml of VEGF showing increased number, size and persistence of F-actin-positive JAIL at the lateral endothelial cell junctions compared to untreated cells. Fluorescence images were taken every 1 second. Elapsed time is designated as mm:ss. Bar, 10 μm.

Movie S13. PKM2 inhibition decreases the number of 'junction-associated intermittent lamellipodia' (JAIL) at the endothelial junctions, Related to Figure 7.

Time-lapse movie of shikonin-treated HUVECs transduced with LifeAct-GFP lentivirus showing diminished F-actin dynamics at the endothelial cell junctions compared to controls. Fluorescence images were taken every 1 minute. Elapsed time is designated as hh:mm. Bar, 10 μm.

Movie S14. PKM2 inhibition decreases the number of 'junction-associated intermittent lamellipodia' (JAIL) at the endothelial junctions, Related to Figure 7.

Time-lapse movie of shikonin-treated HUVECs transduced with LifeAct-GFP lentivirus and treated with 50 ng/ml of VEGF showing reduced number, size and persistence of F-actin-positive JAIL at the lateral endothelial cell junctions compared to controls. Fluorescence images were taken every 1 minute. Elapsed time is designated as hh:mm. Bar, 10 μm.
